# Single-cell Multiomics Analysis of Myelodysplastic Syndromes and Clinical Response to Hypomethylating Therapy

**DOI:** 10.1158/2767-9764.CRC-23-0389

**Published:** 2024-02-12

**Authors:** Ignacio Campillo-Marcos, Marta Casado-Pelaez, Veronica Davalos, Gerardo Ferrer, Caterina Mata, Elisabetta Mereu, Gael Roué, David Valcárcel, Antonieta Molero, Lurdes Zamora, Blanca Xicoy, Laura Palomo, Pamela Acha, Ana Manzanares, Magnus Tobiasson, Eva Hellström-Lindberg, Francesc Solé, Manel Esteller

**Affiliations:** 1 Cancer Epigenetics Group, Josep Carreras Leukaemia Research Institute (IJC), Barcelona, Catalonia, Spain.; 2 Centro de Investigacion Biomedica en Red Cancer (CIBERONC), Madrid, Spain.; 3 Single Cell Unit, Josep Carreras Leukaemia Research Institute (IJC), Badalona, Barcelona, Catalonia, Spain.; 4 Cellular Systems Genomics Group, Josep Carreras Leukaemia Research Institute (IJC), Badalona, Barcelona, Catalonia, Spain.; 5 Lymphoma Translational Group, Josep Carreras Leukaemia Research Institute (IJC), Barcelona, Catalonia, Spain.; 6 Department of Hematology, Experimental Hematology Group, Vall d'Hebron Institute of Oncology (VHIO), University Hospital Vall d'Hebron, Barcelona, Catalonia, Spain.; 7 Department of Hematology, ICO-IJC-Hospital Germans Trias i Pujol, UAB, Badalona, Spain.; 8 Myelodysplastic Syndromes Research Group, Josep Carreras Leukaemia Research Institute (IJC), Barcelona, Catalonia, Spain.; 9 Department of Medicine, Center for Hematology and Regenerative Medicine, Karolinska Institutet, Stockholm, Sweden; Medical Unit Hematology, Karolinska University Hospital, Stockholm, Sweden.; 10 Institucio Catalana de Recerca i Estudis Avançats (ICREA), Barcelona, Catalonia, Spain.; 11 Physiological Sciences Department, School of Medicine and Health Sciences, University of Barcelona (UB), Barcelona, Catalonia, Spain.

## Abstract

Alterations in epigenetic marks, such as DNA methylation, represent a hallmark of cancer that has been successfully exploited for therapy in myeloid malignancies. Hypomethylating agents (HMA), such as azacitidine, have become standard-of-care therapy to treat myelodysplastic syndromes (MDS), myeloid neoplasms that can evolve into acute myeloid leukemia. However, our capacity to identify who will respond to HMAs, and the duration of response, remains limited. To shed light on this question, we have leveraged the unprecedented analytic power of single-cell technologies to simultaneously map the genome and immunoproteome of MDS samples throughout clinical evolution. We were able to chart the architecture and evolution of molecular clones in precious paired bone marrow MDS samples at diagnosis and posttreatment to show that a combined imbalance of specific cell lineages with diverse mutational profiles is associated with the clinical response of patients with MDS to hypomethylating therapy.

**Significance:**

MDS are myeloid clonal hemopathies with a low 5-year survival rate, and approximately half of the cases do not respond to standard HMA therapy. Our innovative single-cell multiomics approach offers valuable biological insights and potential biomarkers associated with the demethylating agent efficacy. It also identifies vulnerabilities that can be targeted using personalized combinations of small drugs and antibodies.

Watch the interview with Manel Esteller, MD, PhD, recipient of the 2026 *Cancer Research Communications* Award for Outstanding Journal Article: https://vimeo.com/1208911530

## Introduction

Myelodysplastic syndromes (MDS) are a group of heterogeneous myeloid clonal hemopathies that cause morphologic bone marrow (BM) dysplasia along with cytopenia and abnormal differentiation, and are associated with an increased risk of transformation to secondary acute myeloid leukemia (AML; refs. 1, 2). With an incidence rate of approximately 4.5 cases per 100,000 people per year in the general population (3) and a median age of diagnosis around 70 years (1, 2), the 5-year survival rate is approximately 37% (2). Clonal hematopoiesis of indeterminate potential (CHIP) associated with the advanced age, also denominated age-related clonal hematopoiesis (4), is thought to be a precursor of MDS (5). However, the underlying mechanisms promoting the progression of CHIP to MDS remain mostly unclear. At the cellular and molecular level, MDS is characterized by different combinations of somatic gene mutations and/or chromosomal abnormalities [such as del(5q)] affecting the myeloid lineage that promote the clonal proliferation of malignant hematopoietic stem cells (HSC). The mutations observed in MDS frequently occur in genes associated with six types of biological functions: DNA damage response (e.g., *TP53*), epigenetic/chromatin modifiers (e.g., *EZH2*), transcription factors (e.g., *RUNX1*), RNA splicing (e.g., *SF3B1*), signal transduction (e.g., *NRAS*), and cohesin complex (e.g., *STAG2*; ref. 6). Some of these genetic events provide prognostic information and, for example, *SF3B1* mutations are usually associated with favorable outcome (7), whereas biallelic loss-of-function mutations in *TP53* are linked to adverse prognosis (8). Recently, the contribution of the BM microenvironment and the inflammatory niche to the natural history of MDS has also been recognized (9) and the first experimental systems studying these surrounding nonhematopoietic cells are starting to be used to understand the biology of MDS (10).

The most used prognostic classifications in MDS are the International Prognostic Scoring System (IPSS; ref. 11) and the revised IPSS (IPSS-R; ref. 12), which take into account the presence/degree of anemia, thrombocytopenia or neutropenia, the percentage of blasts, and karyotypic abnormalities. Very recently, molecular IPSS (IPSS-M) has improved the prognostic discrimination across clinical endpoints (13, 14). If for lower-risk MDS the treatment objective is to ameliorate the symptomatology and no therapy has shown to clearly improve overall survival (OS) in a randomized clinical trial, the first-line therapy for patients with higher-risk MDS are the HMAs (2, 15). HMAs used in MDS include intravenous or subcutaneous azacitidine (AZA), decitabine, or the oral decitabine-cedazuridine. These compounds can act through different pathways including inhibition of DNA methyltransferases, induction of differentiation, and direct cytotoxicity (2, 15). The use of AZA is probably the most implemented and, as most patients with MDS only respond to the compound after several courses, at least six courses are recommended. The use of AZA could yield a median OS of 15–24 months in high-risk MDS (16, 17). The long time required to consider the HMA treatment a failure, associated with the usual 28-day cycles (18) and the overall fragility and co-occurrence of other potential causes of death in these advanced age patients, increase the difficulty to discover biomarkers of clinical response to the HMA treatment, despite the promising cellular and molecular markers that have been proposed (19, 20).

Although (epi)genomic bulk studies in MDS have been critical to leverage the molecular landscape of the disease (13, 14, 21–23), the field was missing a clear picture of the clonal identity and evolution of the disorder that could until recently only be imputed by statistical modeling. However, the irruption of single-cell technologies (24) has given yield to a more granulated view of MDS where clonal mutational architecture can be characterized and the events leading to AML development are starting to be dissected (25–27). Nevertheless, these exciting single-cell recent studies have not particularly addressed how HMA treatment in MDS could shape the evolution of the mutant clones and the immunophenotypes targeted by these genetic events. Most importantly, it has not been explored whether the dynamics of the combined single-cell DNA and cell-surface protein composition of the BM cells upon HMA treatment relates to the clinical response to the epigenetic drug. Here, we provide insights into this unmet medical need by analyzing at the single-cell level the co-occurrence of mutational and cytogenetic events among different cell identities in paired diagnostic and post-HMA treatment BM samples of patients with MDS.

## Materials and Methods

### Patient Samples

Twenty-eight BM samples from 14 patients with MDS were provided by three health care institutions: 8 patients from the collection of samples C.002922 (IJC), 3 from the collection of samples C.0000718 (VHIO), and 3 from the Stockholms medicinska biobank (Karolinska Institute). According to the Biomedical Research Law 14/2007, all patients signed informed consents to donate biological material and clinicopathologic data for research purposes at the reference center, under the approval of the corresponding Ethical Committee. This study has also been approved by the Research Ethics Committee of the Germans Trias i Pujol Hospital (Ref. PI-21-183). BM total cells or bone marrow mononuclear cells (BMMC) from BM aspirates were gathered at two different points: at diagnosis or pretreatment with AZA, and after AZA treatment. BM aspirates or BMMCs were processed following standard operating procedures, cryopreserved and stored in liquid nitrogen until use. Diagnosis and disease status were assigned according to World Health Organization (WHO) 2017 classification (28). Patient characteristics are summarized in Table 1 and the clinicopathologic information of the patients with analyzed MDS, case-by-case, are shown in Supplementary Table S1.

**TABLE 1:** Characteristics of patients with the studied MDS

Characteristics	Number of patients (percentage)
Sex	
Males	9 (64.3%)
Females	5 (35.7%)
Age	
<70 years	7 (50.0%)
≥70 years	7 (50.0%)
MDS WHO 2017 subtypes	
MDS-MLD	3 (21.4%)
MDS-EB1	4 (28.6%)
MDS-EB2	6 (42.9%)
MDS-RS-MLD	1 (7.1%)
Bone marrow blast count at diagnosis	
<5%	4 (28.6%)
5%–9%	4 (28.6%)
10%–19%	6 (42.9%)
IPSS-R cytogenetic risk category	
Very good	0 (0%)
Good	9 (64.3%)
Intermediate	4 (28.6%)
Poor	0 (0%)
Very poor	1 (7.1%)
IPSS-R risk category	
Very low (0 to 1.5)	0 (0%)
Low (2 to 3)	2 (14.3%)
Intermediate (3.5 to 4.5)	2 (14.3%)
High (5 to 6)	7 (50.0%)
Very high (>6)	3 (21.4%)
IPSS-M risk category	
Very low (≤−1.5)	0 (0%)
Low (>−1.5 to −0.5)	0 (0%)
Moderate low (>−0.5 to 0)	1 (7.1%)
Moderate high (>0 to 0.5)	4 (28.6%)
High (>0.5 to 1.5)	5 (35.7%)
Very high (>1.5)	4 (28.6%)
Response to AZA	
Responder	8 (57.1%)
Nonresponder	6 (42.9%)

Abbreviations: IPSS-M, International Prognostic Scoring System-molecular; IPSS-R, International Prognostic Scoring System-revised; MDS, myelodysplastic syndromes; MDS-EB1, MDS with excess blasts type 1; MDS-EB2, MDS with excess blasts type 2; MDS-MLD, MDS with multilineage dysplasia; MDS-RS-MLD, MDS with ring sideroblasts and multilineage dysplasia; WHO, World Health Organization.

Following the International Working Group response criteria in patients with MDS (IWG-MDS; refs. 29–31), a complete response (CR) was defined as BM showing less than 5% blasts with normal maturation of all cell lines with no evidence for dysplasia, and 0% circulating blasts with a hemoglobin level ≥10 g/dL, platelets ≥100*10^9^/L, and neutrophils ≥1.0*10^9^/L. For partial response (PR), patients must demonstrate all CR criteria except that BM blast count >5% and a decrease by 50% or more compared with pretreatment levels. Stable disease was defined as failure to achieve at least PR, but no evidence of progression for >8 weeks. Disease progression was established when at least one of the following criteria was met: (i) In BM and according to the number of blasts of the patient at baseline, for patients with less than 5% blasts at baseline: a 50% or more increase in blasts over baseline to more than 5% blasts; for patients with 5% to <10% blasts at baseline: a 50% or more increase over baseline to more than 10% blasts; and for patients with 10% to <20% blasts at baseline: a 50% or more increase over baseline to more than 20% blasts. (ii) In peripheral blood, a decrease of ≥50% from maximum remission/response levels in neutrophils and neutrophils <1.0*10^9^/L. To evaluate the association between biomarkers and the clinical response, the patients were categorized as responders (CR or PR) and nonresponders (progression or stable disease). Progression-free survival (PFS) was defined as the time from the AZA start to the first either progression to AML or death as a surrogate endpoint.

### Custom DNA and Protein Panels

The custom single-cell DNA sequencing (scDNA-seq) panel designed for this study, which targets 53 commonly mutated genes and recurrently gained or lost regions in patients with MDS (a total of 519 amplicons detailed in Supplementary Table S2), was manufactured by Mission Bio, Inc. The cocktail of oligonucleotide-conjugated antibodies (AOC) targeting 42 cell-surface proteins of interest and three isotype control antibodies (TotalSeq-D Human Heme Oncology Cocktail v1.0; Supplementary Table S3) was purchased from BioLegend.

### Single-cell DNA and Protein Sequencing

scDNA-seq and single-cell protein sequencing (scProt-seq) was performed using the Mission Bio Tapestri platform according to the manufacturer's instructions. Briefly, biological samples were thawed and cells were quantified using the Countess II FL cell counter (Invitrogen, Thermo Fisher Scientific). When required, the “Dead Cell Removal Kit” (Miltenyi Biotec) was employed to fulfill the cell viability criteria recommended by Mission Bio. Next, around 1 million cells were resuspended in Cell Staining Buffer (CSB, BioLegend) and incubated with TruStain FcX (BioLegend) and Cell Blocking (Mission Bio) for 15 minutes at 4°C. The pool of AOCs was then added to the cells and the mix was incubated on ice for 30 minutes. After washing with CSB, cells were resuspended in Cell Buffer (Mission Bio) and counted again. Single cells (3,000–4,500 cells/µL per sample) were encapsulated using a Tapestri microfluidics cartridge, lysed, and barcoded. Targeted DNA regions and antibody–oligonucleotide tags were amplified by incubating the barcoded DNA emulsions in a thermocycler as follows: 98°C for 6 minutes (4°C/second); 11 cycles of 95°C for 30 seconds, 72°C for 10 seconds, 61°C for 9 minutes, 72°C for 20 seconds (1°C/second); 13 cycles of 95°C for 30 seconds, 72°C for 10 seconds, 48°C for 9 minutes, 72°C for 20 seconds (1°C/second); and 72°C for 2 minutes (4°C/second). Emulsions were then broken and DNA PCR products were purified with Ampure XP beads (Beckman Coulter). Protein PCR products (supernatant from Ampure XP beads’ incubation) were incubated with a Biotin Oligo and purified using Streptavidin beads (Mission Bio). Next, DNA and protein PCR products were used as a PCR template for the incorporation of i5/i7 indexes (V2 index primers for DNA libraries and Protein index primers for protein libraries) following this program: 95°C for 3 minutes; 10 cycles (DNA libraries) or 20 cycles (protein libraries) of 98°C for 20 seconds, 62°C for 20 seconds, 72°C for 45 seconds; and 72°C for 2 minutes. Both DNA and protein libraries were purified with Ampure XP beads, quantified and quality checked by using the Qubit fluorometer (Invitrogen, Thermo Fisher Scientific) and the 2200 TapeStation (Agilent). Finally, DNA and protein libraries were subjected to paired-end 150-bp sequencing on a Novaseq 6000 (Illumina).

### Bioinformatic Analysis

#### Preprocessing

FASTQ files were processed using the cloud-based Tapestri Pipeline v.2 (Mission Bio) for adaptor sequence trimming, read alignment to the human genome (GRCh37/hg19) using Burrows-Wheeler Aligner, barcode correction, cell identification, variant calling using GATK and genomic DNA amplicons and antibody-derived tags (ADT) counting. Loom files generated were analyzed using Tapestri Insights v.2.2.

#### Variant Identification

Samples at diagnosis and after AZA treatment of each patient were analyzed jointly with Tapestri Insights using the following filters to retain high-quality genotype calls: genotypes with quality >30, read depth >10 and alternate allele frequency >20%. In addition, only variants genotyped in >50% of cells were kept for subsequent analysis, with the exception to those exhibiting good quality and alternate allele frequency, which were reported in the analysis, but not included as a single clone. Variants mutated in <1% of cells and cells with <50% of genotypes present were discarded. Variants reported with bulk targeted next-generation sequencing (NGS) were included as a whitelist in the analysis. Finally, variants with minor allele frequency >0.01, according to population databases, were filtered out. Exonic (nonsynonymous single-nucleotide variants and indels) and splicing variants were retained for the analysis. These variants were then assessed for pathogenicity using ClinVar and Varsome databases (32, 33). To determine the allele dropout (ADO) rate, variants mutated in ≥92% cells at both timepoints and whose variant allele frequency (VAF) percentage by cell count were close to 50% (from 49.2% to 52.7%) were selected (27). The ADO of each sample was calculated by applying the formula: [(number of wild-type (WT) cells + number of homozygous cells)/total number of genotyped cells] × 100, in five germline heterozygous variants, and computing the average (27).

#### Clone Identification

Cell clones were defined by clustering cells based on the previously selected variants after quality control using Tapestri Insights. A clone was defined by copy-number variation (CNV) alterations when deletions or insertions were observed by inspecting changes in the VAF of a heterozygous variant in the same chromosome region. CNV clones were validated with Mosaic package (v.2.0.4). ADO clones were manually inspected and discarded. Finally, clonal phylogenies were reconstructed on the basis of genotype clustering, zygosity information, and co-occurrence of mutations using the diagnosis and AZA treatment samples of each patient (27). A total of 109,507 cells retained after variant and genotype quality control were used for analysis that only comprise DNA information.

#### Ploidy Computation

Generated H5 files from Tapestri Pipeline were analyzed using python-based Mosaic package (v.2.0.4). Clones identified in Tapestri Insights were reproduced in Mosaic providing as whitelist the previously selected variants and applying *group_by_genotype* function. For the CNV analysis, amplicons found in less than half the total cells were discarded. To correct cell to cell and amplicon to amplicon variations, normalization was performed using *normalize_reads* function. Cells and amplicons were normalized on the basis of their total reads and their mean counts, respectively. The ploidy was computed using *compute_ploidy* function, which requires the barcodes of the clone known to be diploid for baseline correction. WT cells were considered as reference of diploidy to identify copy gain and losses in mutant clones per sample.

#### Protein Analysis

ADT counts from H5 files were extracted using Mosaic and analyzed in R (v.4.2.2). For the protein analysis, cells with <200 and >100,000 ADT counts and cells belonging to clusters exhibiting homogeneous abundances across all antibodies (likely by technical artefacts) were discarded. Thus, from the 109,507 cells, only 90,721 cells passed the quality control for the protein analysis. Scaling using count per million with a +1 pseudocount and centered-log ratio (CLR) normalization were applied to ADT counts, followed by cell-to-cell noise removal using control isotypes as background noise (34, 35, bioRxiv 2022.08.25.505316). The normalized matrix was analyzed with Seurat package (36). Principal component analysis was applied to the normalized matrix to perform dimensionality reduction. Subsequently, shared nearest neighbor graph was generated using k-nearest neighbors applying Louvain algorithm to cluster the cells. Clusters were explored in further detail using *FindSubCluster* function. For clustering visualization, Uniform Manifold Approximation and Projection (UMAP) was applied. To assess the integration of all samples, we used the local inverse Simpson index (LISI; ref. 37). This index indicates the effective number of samples in the local neighborhood of a cell. A LISI score near the total number of samples represents a good mixing across patients and timepoints, indicating that no cluster is uniquely driven by the effect of a single sample.

#### Cell Type Annotation

To annotate cell populations, cell-type specific protein markers were inspected. Clusters exhibiting homogeneous abundances across all antibodies, likely by technical artefacts, were discarded. Of note, four rare cell populations were defined by the expressed surface markers, but only one of them (CD11c+ CD45+ CD49d+ CD62P+ cells) was considered after cell type compositional analysis, because the other three populations were predominantly found in only one sample. Finally, the annotation was refined by separately analyzing lymphoid, myeloid, and progenitor compartments. The four rare populations were not included in any of the major compartments [progenitors, immature erythroid cells, myeloid cells, T cells, natural killer (NK) cells, and B cells].

#### Cell Type Compositional Analysis

We applied scCODA (38), a Bayesian model tailored to conduct compositional analysis of single-cell data, to identify statistically significant changes in cell type abundance between conditions (timepoint or response). For each test, we set a False Discovery Rate (FDR) threshold of 0.1 (39, 40) and applied an automatic reference selection (38).

#### Figure Generation and Statistical Analysis

Figures were generated in R (v.4.2.2) using ggplot2 (v.3.4.2), ggraph (v.2.1.0), TimeScape (v.1.22.0), ComplexHeatmap (v.2.14.0), and ComplexUpset (v.1.3.5) R packages and Biorender. Comparisons of numerical variables according to timepoint or response state were computed using the Wilcoxon rank-sum test (using paired test for timepoint and unpaired test for response). Associations between two categorical variables were assessed with two-sided Fisher test. Shannon diversity index was computed using the vegan R package (v.2.6-4). Spearman test was applied to evaluate the correlation between single-cell and bulk VAFs. *P* values were adjusted for multiple comparisons using Bonferroni correction when multiple tests were carried out. Multivariate Cox hazards regression analyses were performed for the study of PFS.

### Data Availability

scDNA-seq and scProt-seq data have been deposited in European Genome-Phenome Archive (EGA) at https://ega-archive.org/studies/EGAS00001007427 (accession number EGAS00001007427).

## Results

### Single-cell Genetic Landscape of the MDS Cases Treated with HMA

**FIGURE 1:**
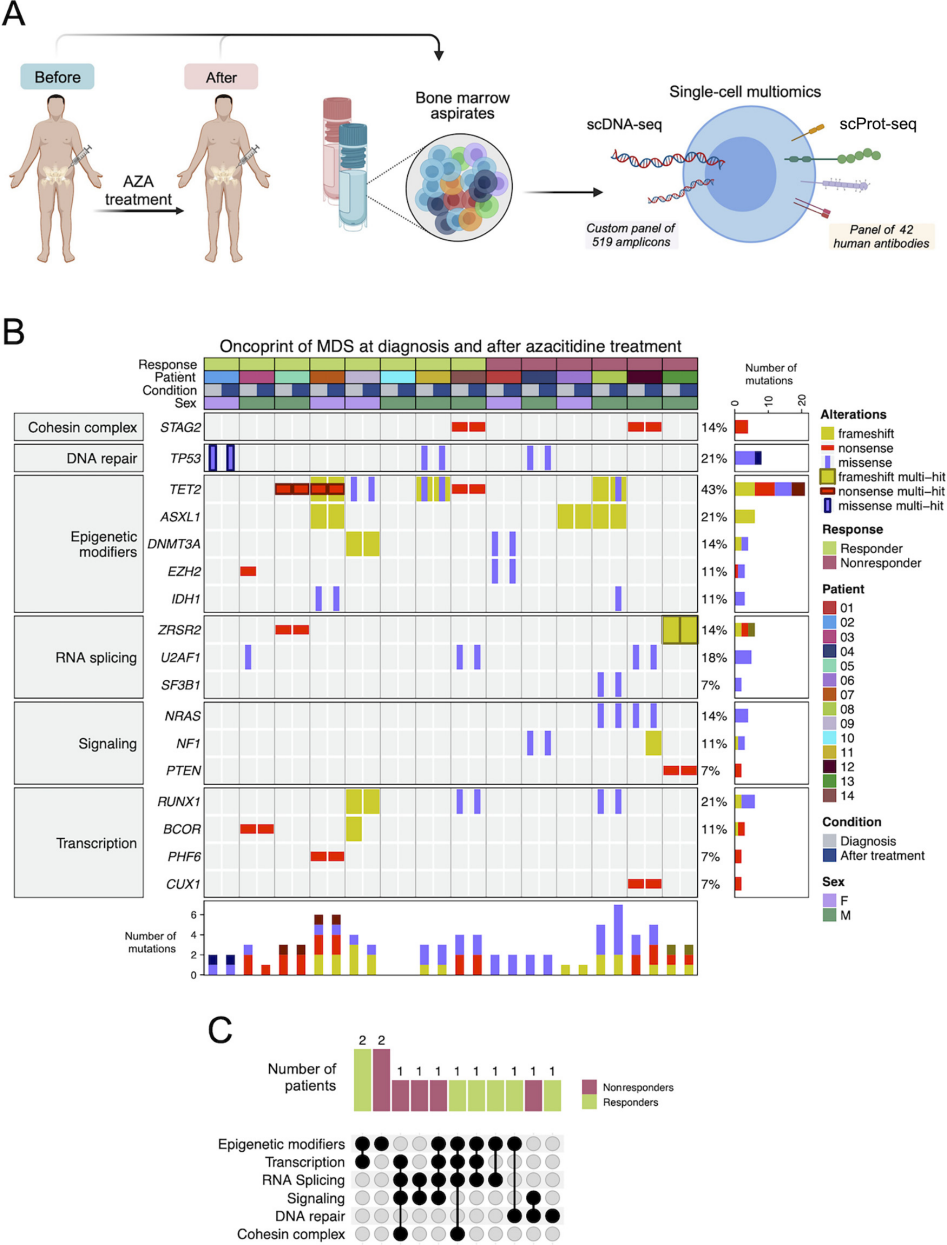
Mutational landscape of patients with the studied MDS. **A,** Summary of the study workflow. **B,** Oncoprint indicating the mutations present in the patient cohort, at diagnosis and after AZA treatment, colored by coding impact. Multi-hit means presence of more than one mutation of same coding impact (missense, nonsense, or frameshift) in the same gene. **C,** UpSet plot illustrating the exclusive intersection of mutated pathways at diagnosis in patients with MDS. The patients are colored according to the response status (green, responders; magenta, nonresponders).

To study the evolution of the clonal architecture of MDS upon the treatment with HMA, we performed scDNA-seq, using a 519 custom amplicon panel, to study 53 genes commonly mutated in myeloid malignancies (Supplementary Table S2) and scProt-seq of a panel of 42 cell-surface proteins (Supplementary Table S3), including principal lineage antigens, to provide a simultaneous landscape of the genetic setting and immunophenotype at single-cell resolution (Fig. 1A). We sequenced 90,721 cells from 28 samples corresponding to 14 patients with MDS where paired BM samples were obtained at diagnosis and after AZA treatment (Fig. 1A). All MDS cases were classified according to the IPSS-R risk group (ref. 12; Table 1). Following the IWG-MDS (29–31), HMA treatment induced a clinical response in eight cases (57.14%) and the nonresponders were 6 patients (42.86%). The clinicopathologic characteristics of the analyzed MDS cases are shown in Table 1 and case-by-case in Supplementary Table S1. The mutated genes identified with scDNA-seq at diagnosis timepoint were: *TET2* (6/14, 42.86%); *ASXL1*, *RUNX1*, *TP53*, and *U2AF1* (3/14, 21.43%); *BCOR*, *DNMT3A*, *EZH2*, *ZRSR2*, *STAG2*, and *NRAS* (2/14, 14.29%); and *IDH1*, *PHF6*, *PTEN*, *NF1*, *CUX1*, and *SF3B1* (1/14, 7.14%; Fig. 1B). The same mutations were observed in the post-HMA samples except in 4 patients: 2 of them showed acquisition of new mutations (*NF1* and *IDH1/TET2*) and the remaining 2 patients lost *BCOR* or *EZH2/U2AF1* mutant cells (Fig. 1B). The characteristics of the identified mutations in the interrogated MDS cases are shown in Fig. 1B. The ADO rates per sample (diagnosis and after AZA treatment) are shown in Supplementary Table S4. We observed that the occurrence of any individual mutation for the studied genes was not associated with the clinical response to the HMA (for all cases Fisher exact test, adjusted *P* value >0.05; Supplementary Table S5). The total number of accumulated mutations in each patient was also not associated with the efficacy of the HMA therapy (Supplementary Fig. S1A). The reconstruction of common VAF from the scDNA-seq data correlated significantly with bulk NGS (Spearman correlation test *R* = 0.88; *P* <1.6 e^−09^; Supplementary Fig. S1B). Supplementary Table S6 shows the karyotype, NGS, and scDNA-seq data at diagnosis for each patient.

We investigated the single-cell mutational profile and its potential impact in the clinical response to HMA therapy in a two-step manner. First, only considering the genetic setting at diagnosis; and second, analyzing the evolution of the mutational clonal spectrum upon treatment with the demethylating agent. At disease presentation, 92.86% of MDS cases (13/14) had a coding mutation in at least one gene included in the six commonly mutated pathways: epigenetics (9/14, 64.29%), including DNA methylation-related genes *DNMT3A*, *TET2*, and *IDH1* (7/14, 50%) and chromatin/histone modifiers *EZH2* and *ASXL1* (5/14, 35.71%); transcription factors (6/14, 42.86%); RNA splicing (6/14, 42.86%); signaling genes (4/14, 28.57%); *TP53* (3/14, 21.43%) as example of DNA damage response, and *STAG2* as example of the cohesin complex (2/14, 14.29%; Fig. 1B). Interestingly, if mutations for different genes in the same pathway were observed for the epigenetics, transcription factors, and signaling categories, the simultaneous presence of two mutations were never found in splicing genes (Fig. 1B). The distribution of the mutations among the studied pathways was not associated with response to HMA treatment (for all cases Fisher exact test, adjusted *P* value >0.05; Supplementary Table S7). The combination of mutated pathways in the patients with studied MDS is shown in Fig. 1C. None of these combinations were associated with HMA response (for all cases, Fisher exact test, adjusted *P* value >0.05; Supplementary Table S8).

**FIGURE 2:**
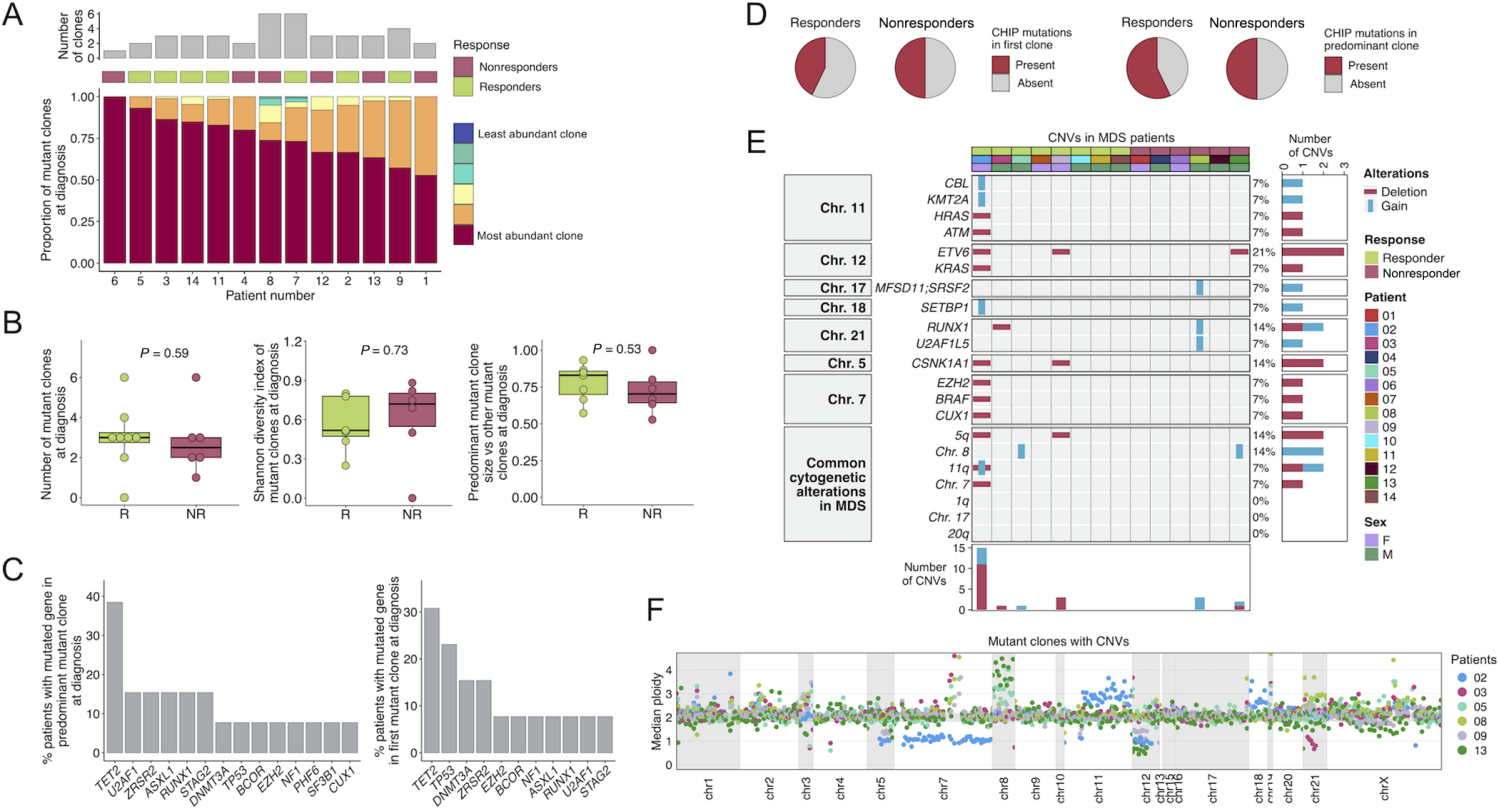
Distribution of mutant clones and CNVs at diagnosis in patients with the studied MDS. **A,** Proportion of mutant clones over all mutant cells at diagnosis in each patient, colored by clone abundance. Upper bar plot illustrates number of clones at diagnosis and the middle bar indicates the response status (green, responders; magenta, nonresponders). Note that patient #10 is not included because no mutant clones were found using our gene panel. **B,** Number of mutant clones at diagnosis (left); Shannon diversity index computed among mutant clones at diagnosis (middle); predominant mutant clone size with respect to other mutant clones at diagnosis (right). R, responders; NR, nonresponders. **C,** Percentage of patients with mutations in each gene in the predominant clone at diagnosis (left); Percentage of patients with mutations in each gene in the first clone at diagnosis (right). **D,** Proportion of responders and nonresponder patients with CHIP mutations (*TET2, DNMT3A*, and *ASXL1*) in first (left) and predominant (right) clones at diagnosis. **E,** Oncoprint of CNVs in the patient cohort at diagnosis. **F,** Median per amplicon ploidy of the mutant clones for patients with CNVs (patients #2, #3, #5, #8, #9, and #13).

We next investigated the clonal mutational distribution of the MDS cases at diagnosis, with clones defined as cells with identical mutations. At the level of clone resolution provided by the genetic variants above described for the 53 genes, we found that for most of the patients with mutated MDS (92.31%, 12/13) different molecular clones were already present at the disease presentation stage. Among all cases, the number of mutant clones ranged from 1 to 6 with an average of 3.15 clones per MDS sample. The abundance of each mutant clone in each patient is also illustrated in Fig. 2A. The crude number of individual mutational clones in a given MDS sample at diagnosis was not associated with the clinical response to HMA (Wilcoxon test, *P* = 0.59; Fig. 2B). We also studied the mutant clone diversity on a per-sample basis and did not observe significant differences between HMA responders and nonresponders (Shannon diversity index, Wilcoxon test, *P* = 0.73; Fig. 2B). Finally, we did not find an association between the relative size of the largest mutant clone and the clinical response to HMA (Wilcoxon test, *P* = 0.53; Fig. 2B).

From a qualitative perspective, we assessed whether particular mutated genes were more likely to appear in the predominant MDS mutant clone and its relationship with HMA response. Mutated *TET2* gene was the most frequent component of the predominant MDS clone, occurring in 38.46% of MDS mutated cases (5/13; Fig. 2C). The presence of *TET2* in the mutant predominant clone, or any other particular gene, was not associated with clinical response to HMA (for all cases, Fisher exact test, *P* > 0.05). scDNA-seq provided the opportunity to trace the trajectory of somatic events leading to the observed mutant clones in the MDS diagnosis sample. In agreement with its common presence in the most abundant mutant clone (Fig. 2C), we observed that *TET2* was the most frequently mutated gene present in the initial clone (4/13, 30.77%; Fig. 2C). Interestingly, when we analyzed the type of mutation occurring in the original clone, 46.15% (6/13) of the MDS started with an event in a gene related to expression regulation, such as epigenetic (*TET2* and *DNMT3A*) or transcription factor (*BCOR*) functional categories. Related to CHIP defined by the occurrence of mutations at the *TET2*, *DNMT3A*, or *ASXL1* genes, we observed that this event was present in the first mutant clone in 46.15% (6/13) of the studied MDS cases without any association with the clinical response to HMA (Fisher exact test, *P* > 0.05; Fig. 2D). The described CHIP phenotype was found in the predominant MDS mutant clone in 53.85% (7/13) of cases without any association with the response to the demethylating drug treatment (Fisher exact test, *P* > 0.05; Fig. 2D). We also performed these analyses by considering CHIP as the occurrence of mutations also in *SF3B1* and *TP53* (in addition to *TET2*, *DNMT3A*, and *ASXL1*) or defined by the presence of mutations in any leukemia driver gene at a VAF of >2% (Supplementary Fig. S2). None of these definitions of CHIP in the first clone or in the predominant clone were associated with the clinical response to HMA (for all cases Fisher exact test, *P* > 0.05; Supplementary Fig. S2).

scDNA-seq was also used to determine CNVs not only for genes included in the custom panel for mutation detection, but also for genomic regions commonly affected by CNV in MDS such as both arms of chromosomes 7, 8, and 17 and the long arms (q) of chromosomes 1, 5, 11, and 20 (41). A CNV oncoprint indicating the presence of deletion or gain in the described loci for all the studied 14 patients with MDS is shown in Fig. 2E. The most commonly deleted gene was the transcription factor *ETV6* (3/14, 21.43%), and its loss was not associated with HMA response (Fisher exact test, *P* > 0.05). The number of CNV events was evenly distributed among cases, except for the patient #2 (a responder to HMA therapy) that accumulated many more CNV alterations (Fig. 2E). The median per-amplicon ploidy of the mutant clones for the patients with CNVs is shown in Fig. 2F.

**FIGURE 3:**
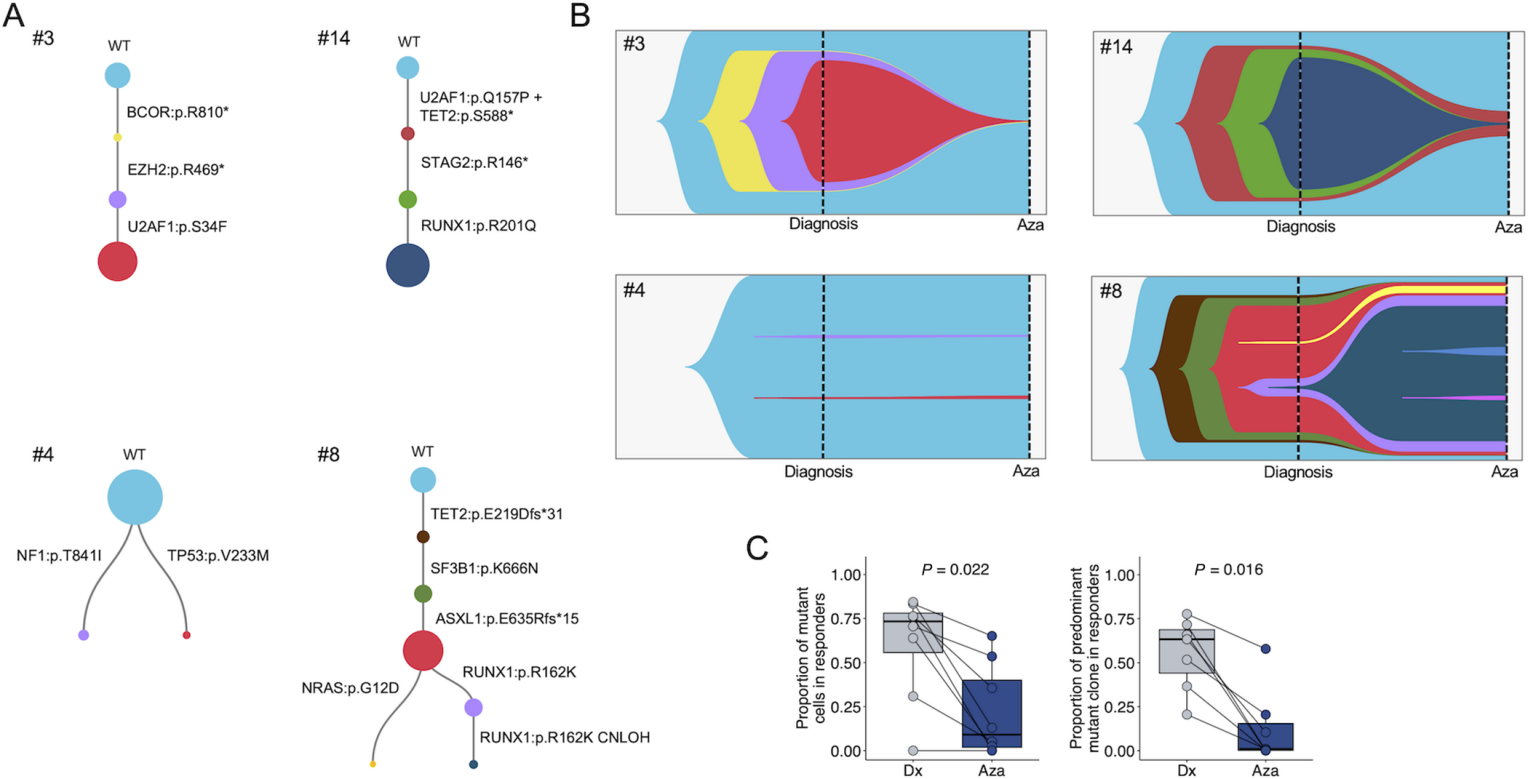
Clonal evolution of responder and nonresponder patients with MDS upon AZA treatment. **A,** Clonal phylogenies of patients #3 and #14 (responders) and #4 and #8 (nonresponders) at diagnosis. Dot size represents clone size. **B,** Fishplots of patients #3 and #14 (responders) and #4 and #8 (nonresponders), illustrating the clonal distribution at diagnosis and after AZA treatment. **C,** Proportion of mutant cells (left) and predominant mutant clone (right) in responder patients at diagnosis and after AZA treatment. Dx, diagnosis; Aza, after AZA treatment.

We proceeded with the trajectory phylogeny of the clones at the diagnosis timepoint according to the mutational composition, using the same method for similar scDNA-seq Tapestri analysis recently reported in MDS cases to study their progression to AML (27). Related to the reconstruction of clonal evolution, we observed in 61.54% of cases (8/13) a branching evolution pattern of mutational events, whereas in 38.46% (5/13) of MDS mutated cases a linear phylogeny with a consecutive acquisition of genetic mutations was observed. Interestingly, in the cases with branched architecture, all cases except two (patients #1 and #4) showed the predominance of a clone that indicates cellular fitness of that population, whereas in these two cases the relative weight of each mutant population was more equally distributed (Fig. 3A; Supplementary Fig. S3), suggesting a more neutral branching evolution (42). The type of trajectory phylogeny (linear or branched) was not associated with any particular mutant gene or gene pathway (Fisher exact test, *P* > 0.05). Importantly, the presence of a linear or branched evolution pattern was not significantly associated with clinical response to HMA (Fisher exact test, *P* = 0.27). The obtained phylogenies for all cases are shown in Fig. 3A and in Supplementary Fig. S3.

We next proceeded to delineate whether the characterized scDNA-seq profiles underwent changes in response to HMA therapy. Assessing the clonal mutational architecture of the initial MDS diagnosis sample with its paired counterpart after HMA treatment, we observed three main clonal evolution patterns (Fig. 3B; Supplementary Fig. S3). The first profile is characterized by the reduction of the size of the different mutant clones upon HMA therapy and its bottle-neck convergence in identical clones for mutational repertoire but with much lower number of cells (e.g., patients #3 and #14 in Fig. 3B). The diminishment of the mutant clones upon HMA treatment in the responders included the predominant clone, which is the one likely to harbor a higher cellular fitness, but also other mutant clones (Fig. 3B). This pattern was observed in all (7/7) of the patients with MDS that achieved a clinical response to the HMA treatment. Patient #10 was not included because no mutant clones were found using our gene panel. The other two types of clonal dynamics were characterized either by the absence of any significant effect in any mutant clone size upon the administration of the HMA (observed in five cases; e.g., patient #4 in Fig. 3B), or by the expansion of initially minority mutant clones and the appearance of new mutant clones not observed in the MDS diagnosis sample (only one case, patient #8 in Fig. 3B). These last two clonal evolution patterns were observed in all (6/6) the patients with MDS that exhibited a lack of response to the HMA therapy (Fisher exact test, *P* < 0.001). These different clonal evolutions patterns were not associated with PFS according to multivariate Cox hazards regression analyses (*P* > 0.05). The fishplots illustrating the clonal distribution for all patients are shown in Fig. 3B and in Supplementary Fig. S3. Thus, overall, the shrinkage of the proportion of mutant clones to yield a few surviving mutant cells upon HMA treatment was associated with the patients that underwent clinical response (Wilcoxon test, *P* = 0.022; Fig. 3C). In a similar manner, the reduction of the predominant mutant clone upon HMA therapy was also associated with clinical response to the epigenetic drug (Wilcoxon test, *P* = 0.016; Fig. 3C).

### Single-cell Protein Immunophenotyping of Patients with MDS Upon HMA Therapy

**FIGURE 4:**
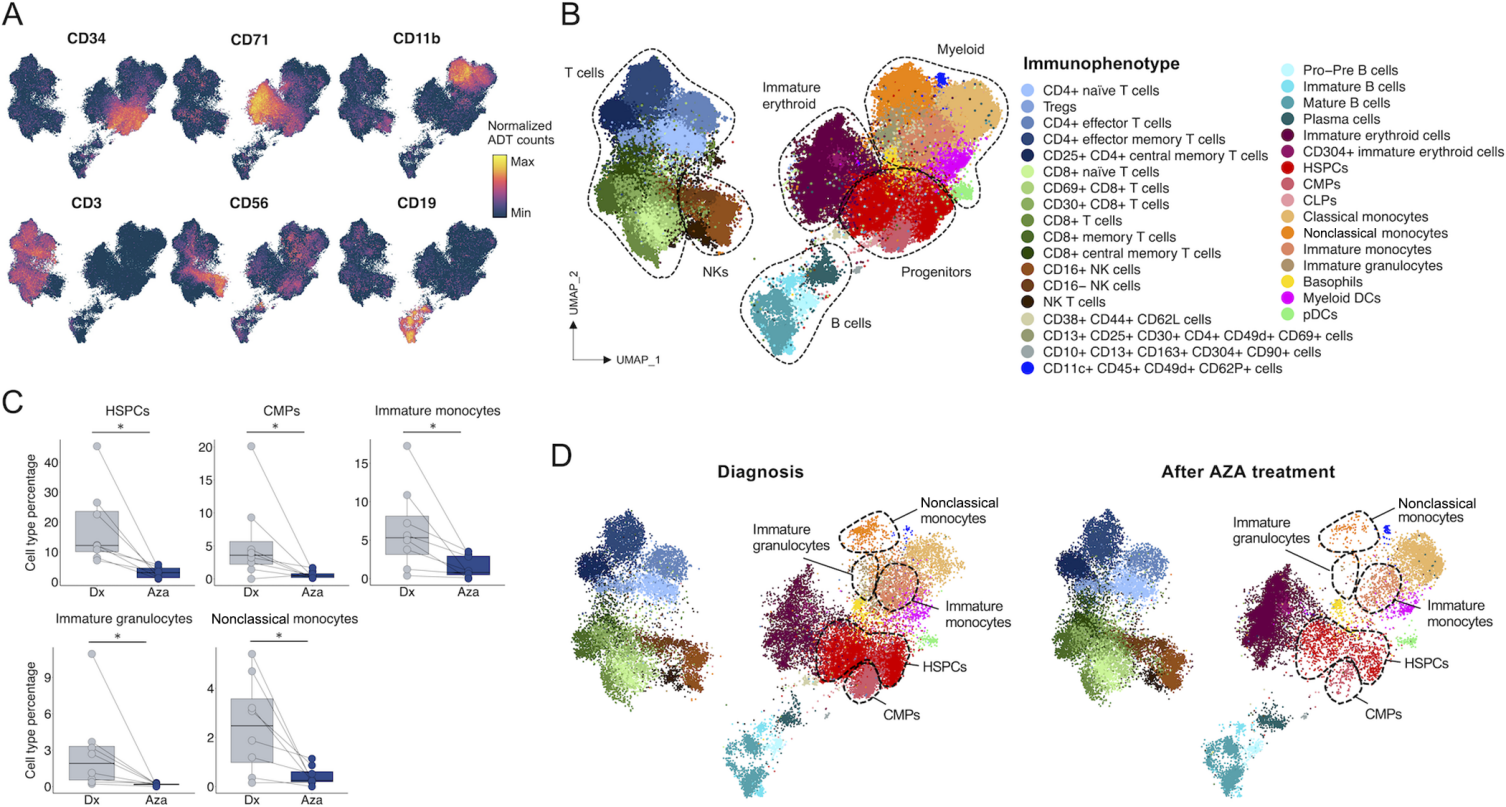
Characterization of BM cell populations of patients with MDS based on scProt-seq data. **A,** UMAP of BM cells from all MDS samples colored by abundance of the depicted proteins. **B,** UMAP of BM cells from all MDS samples colored by cell type annotation. **C,** Cell type proportions at diagnosis and after AZA treatment in responders. *, scCODA FDR <0.1. **D,** UMAPs at diagnosis and after AZA for all the responder patients. Dx, diagnosis; Aza, after AZA treatment.

We next sought to investigate whether single-cell protein data provided insights about the cellular processes taking place under HMA treatment in MDS and its possible value as candidate biomarkers of clinical response to the drug. Using the Mission Bio Tapestri approach with oligo-tagged protein antibodies for this aim (43), we characterized the single-cell immunophenotype determined by 42 cell-surface proteins (Supplementary Table S3) in our paired MDS samples at diagnosis and after the treatment with AZA. UMAP plots showing cells colored by all the studied samples are shown in Supplementary Fig. S4A. UMAPs colored by the LISI score (37), an index to interrogate the presence of batch effect, showed that all cells were well-mixed across all MDS samples (Supplementary Fig. S4B). UMAPs using the standard specific cell-surface markers for the progenitor (CD34), immature erythroid (CD71), myeloid (CD11b), T-cell (CD3), NK cell (CD56), and B-cell (CD19) lineages were able to capture these cellular compartments in our patients with MDS (Fig. 4A). A detailed analysis of the single-cell protein data for all integrated samples was able to visualize 34 cell populations (Fig. 4B) according to its antibody labeling (dot-plot in Supplementary Fig. S4C). We then proceeded to identify a possible uneven distribution of the identified 34 immunophenotypes according to the clinical response to HMA in the patients with MDS at diagnosis, after HMA treatment or comparing both timepoints. To detect changes in cell type abundances between conditions (timepoint or response) while controlling for FDR, we have applied scCODA (38), a Bayesian model specifically tailored for compositional analysis of single-cell data (Materials and Methods). We were not able to identify any cell type population associated with HMA response at the time of diagnosis (scCODA FDR >0.1). In contrast, when comparing the samples collected post-HMA treatment, three cell populations were significantly different between responders and nonresponders. We observed that nonclassical monocytes (scCODA FDR <0.1) were enriched in those patients that did not respond to the HMA therapy (Supplementary Fig. S5A); whereas immature erythroid and mature B cells (both scCODA FDR <0.1) were depleted in HMA nonresponder patients (Supplementary Fig. S5A). All these populations are highlighted in the UMAPs of the Supplementary Fig. S5B.

As mentioned above, the final comparison between the content of the identified 34 cell populations was established between their values at the paired MDS samples at diagnosis versus the levels detected following the cycles of HMA treatment. This consecutive pre- and post-HMA approach was the most successful and we found five cell populations that were significantly associated with HMA clinical response. Those patients that responded to the HMA therapy were characterized by a post-HMA depletion of two progenitor cell populations: hematopoietic stem and progenitor cells (HSPC) and common myeloid progenitors (CMPs; for both cell populations scCODA FDR <0.1; Fig. 4C). In the myeloid lineage, these HMA responders underwent a depletion of immature monocytes, immature granulocytes and nonclassical monocytes (for all cases, scCODA FDR <0.1; Fig. 4C). None of these cell types was associated with PFS according to multivariate Cox hazards regression analyses (*P* > 0.05). The UMAPs derived from all responder patients with MDS, at diagnosis and post-AZA, are shown in Fig. 4D. Illustrative examples for individual patients are depicted in Supplementary Fig. S5C. Interestingly, among these five populations associated with clinical response to HMA, the depletion of HSPCs and immature monocytes was also associated with hematologic improvement (HI; scCODA FDR <0.1; Supplementary Fig. S6A); and the reduction of HSPCs was also linked to marrow complete remission (mCR; Supplementary Fig. S6B).

### Combined scDNA-seq and Immunophenotyping of Patients with MDS Upon HMA Therapy

**FIGURE 5:**
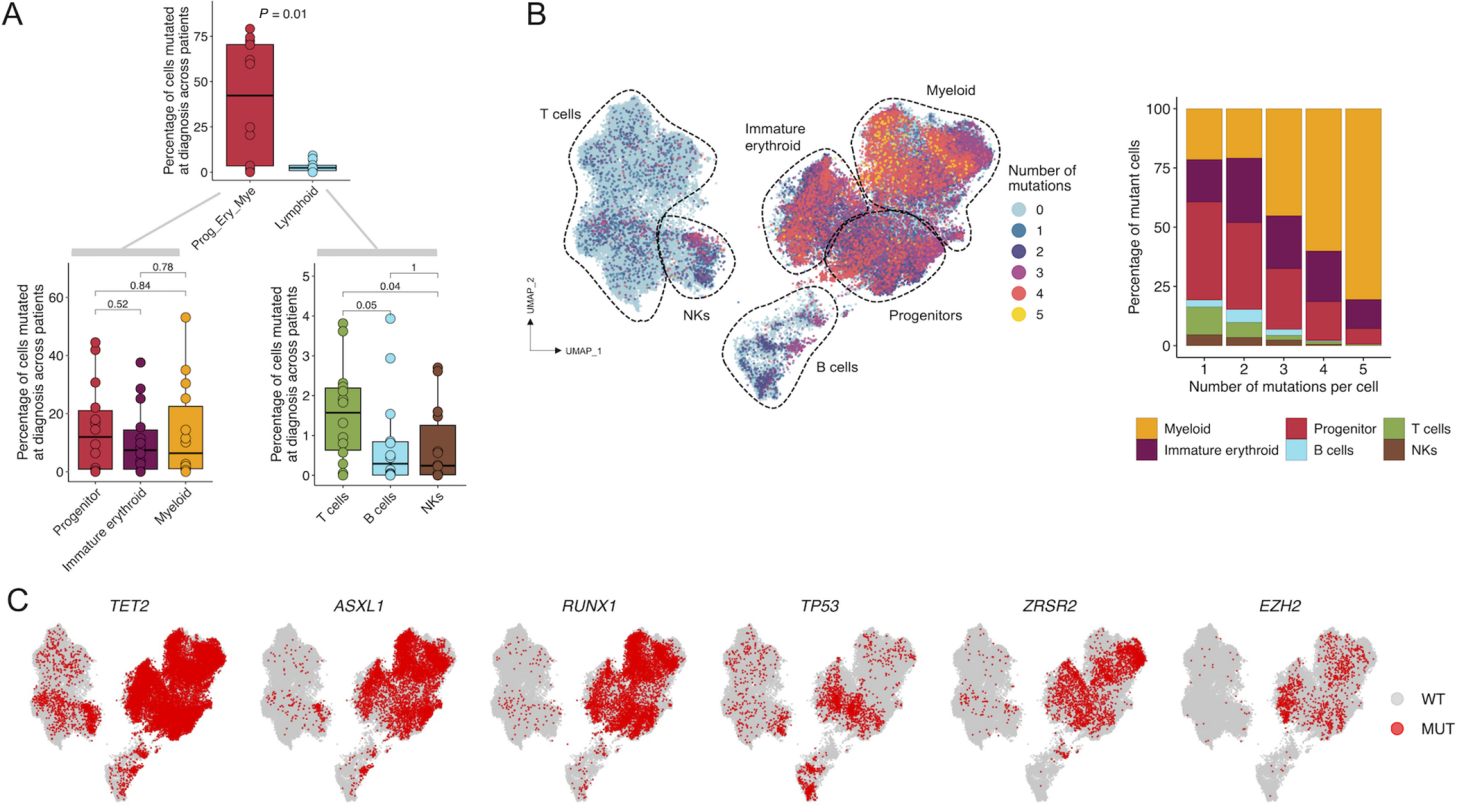
Distribution of mutant cells within the BM compartments defined by the immunophenotype. **A,** Percentage of mutant progenitor, immature erythroid and myeloid cells (Pro_Ery_Mye) compared with mutant lymphoid cells in each patient at diagnosis (top); percentage of mutant progenitor, immature erythroid and myeloid (bottom, left); or T, B, and NK cells (bottom, right) in each patient at diagnosis. **B,** UMAP of BM cells from all samples colored by number of mutations per cell (left); cell type proportions according to the number of mutations per cell (right). **C,** UMAP colored according to gene mutational status. WT, wild-type; MUT, mutant.

**FIGURE 6:**
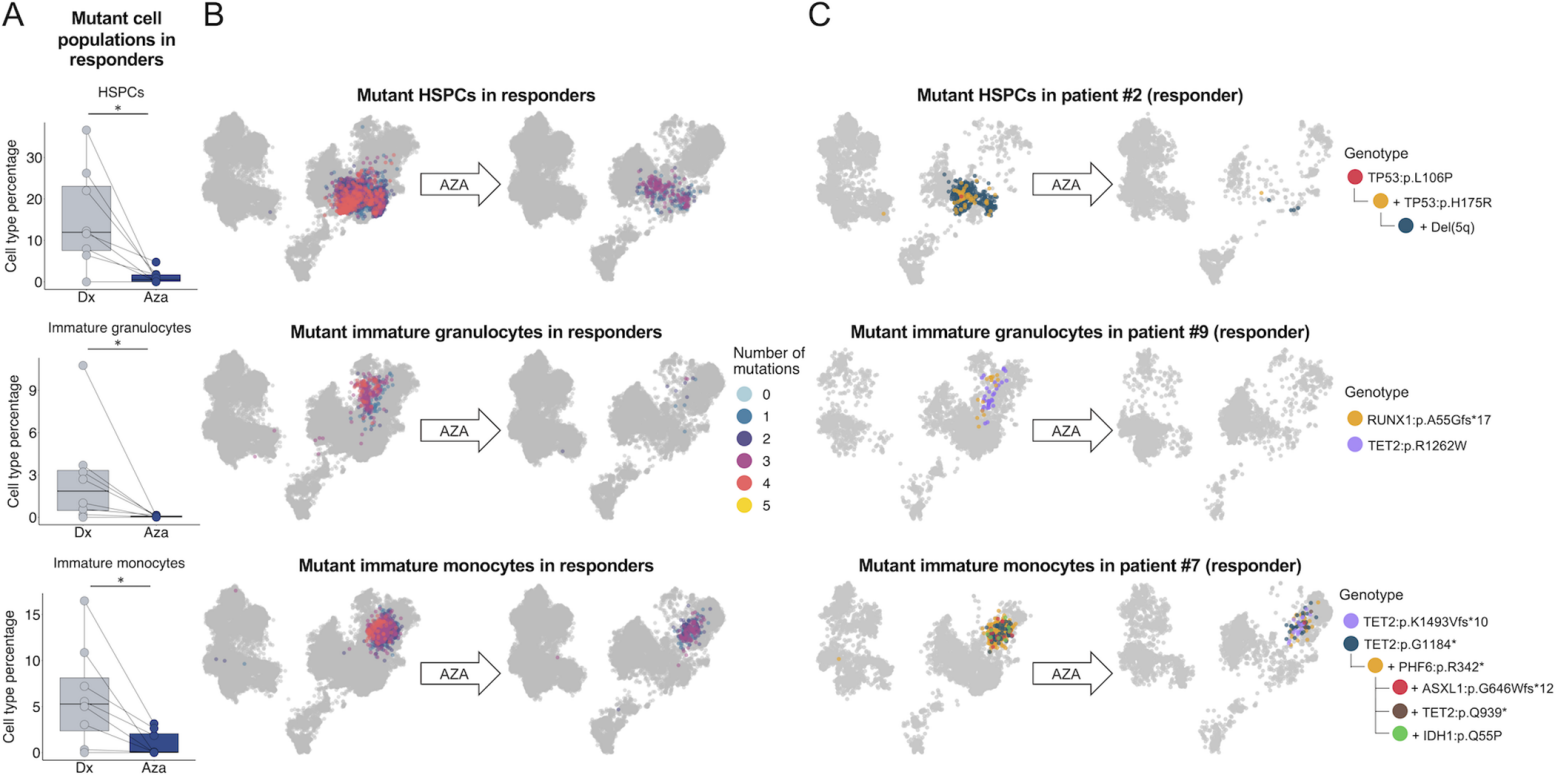
Impact of AZA treatment on mutant cell populations in responder patients. **A,** Mutant cell type proportions at diagnosis and after AZA treatment in responders. *, scCODA FDR <0.1. **B,** UMAPs at diagnosis and after AZA in all the HMA responder patients, colored by number of mutations per cell, highlight the three mutant populations significantly associated with response. **C,** UMAPs highlighting mutant HSPCs in patient #2 (responder), mutant immature granulocytes in patient #9 (responder) and mutant immature monocytes in patient #7 (responder), colored by clone. Dx, diagnosis; Aza, after AZA treatment.

We next wondered whether the MDS mutational landscape identified at the single-cell level was associated with any particular cell type and immunophenotype derived from the single-cell protein approach described above, and whether there were particular shifts of the combined genotype-antibody profiles upon HMA treatment. Among all the mutations identified in MDS cases at the diagnosis stage, we found overall a predominance of the mutant cells versus WT cells at the progenitor, immature erythroid and myeloid populations (as defined in Fig. 4B) in comparison to the lymphoid lineage (Wilcoxon test, *P* = 0.01; Fig. 5A). Among the progenitor, immature erythroid and myeloid compartments, the number of mutant cells were evenly distributed (Wilcoxon test, *P* > 0.05; Fig. 5A). The clonal landscape of the variants in progenitor, immature erythroid, myeloid, and lymphoid compartments for each patient is detailed in Supplementary Fig. S7. Lymphoid cells may harbor mutations detected in myeloid cells indicating the contribution of mutant stem cell clones still capable of multilineage output in CHIP, as described previously (27). For those few mutant cells present in the lymphoid lineage, we observed a higher percentage of mutant T cells compared with mutant B and NK cells (Fig. 5A). Beyond the proportion of mutated cells by population described above at diagnosis, the accumulation of mutations (from zero to five) in a given unique cell according to its immunophenotype in all paired samples also demonstrated an enrichment in the progenitor, immature erythroid and myeloid populations in comparison to the lymphoid lineage, as illustrated in the UMAP of Fig. 5B. Classifying by specific mutated genes, we observed that most of the identified mutated genes (94.12%, 16/17) followed this overrepresentation of mutated forms in the progenitor, immature erythroid and myeloid cell types (Fisher exact test, adjusted *P* value <0.05; Supplementary Table S9). The corresponding UMAPs are shown in Fig. 5C and Supplementary Fig. S8A. The only exception was the DNMT3A missense mutation p.V636M detected in patient #1 that was enriched in the lymphoid lineage (Supplementary Fig. S8B), particularly in the T-cell lineage (over 90% of mutated cells, Fisher exact test, *P* = 2.20 e^−16^). Interestingly, non-R882 DNMT3A mutations (such as the one herein shown) have been previously described to be overrepresented in T cells in MDS compared with the R882 DNMT3A mutation that is enriched in the myeloid lineage (25). The observed DNMT3A V636M, however, is of uncertain significance such it has only been found once in the MDS samples currently available in cBioPortal. Regarding mutations in the lymphoid lineage at diagnosis, mutant cells for each gene were very rare (<5.3% of all lymphoid cells considering all patients with MDS) and mostly occurred in CHIP genes in our MDS-focused panel (Supplementary Fig. S9). Finally, we applied the combinatorial approach of scDNA-seq and scProt-seq to seek immunogenetic phenotypes associated with the clinical response to HMA in MDS. In this regard, for the identified five cellular populations that were associated with HMA response in the diagnosis versus post-HMA treatment comparison described above (Fig. 4C), we subdivided them in those that carry mutant genes versus those that only harbor WT sequences for the studied genes. For these five populations, three (60%) remained associated with HMA response according to their mutational status. These three mutant populations were not associated with PFS according to multivariate Cox hazards regression analyses (*P* > 0.05). For all these three cases, we found that only the depletion in the post-HMA sample of the mutant HSPCs, immature granulocytes, and immature monocytes (for all cases, scCODA FDR <0.1) was associated with HMA response (Fig. 6A), whereas no significant differences were found in their counterpart WT populations (Supplementary Fig. S10). UMAPs of all HMA responder patients highlight how mutant cells of these three populations are significantly reduced after AZA treatment, especially those that harbor a higher mutational load (Fig. 6B). Illustrative examples of the mutant populations in 3 HMA responder patients with MDS are shown in Fig. 6C. Interestingly, among the three mutant populations associated with HMA response, the depletion in the post-HMA sample of the mutant HSPCs was also linked to HI (scCODA FDR <0.1; Supplementary Fig. S11).

## Discussion

MDS are common hematologic malignancies that predominate in the elderly, with a median age of diagnosis of 72 years (44, 45). Most of the affected individuals will die from the disorder-related clinical complications such as cytopenia or worsening of previous comorbidities. Importantly, around 30%–40% of MDS will at one point transform into an AML that shows commonly a poor clinical outcome. The increase of an aged population in the Western countries indicates that the efficient handling of MDS is going to be an important medical necessity in the coming years. In patients with higher-risk MDS, the treatment with HMAs like AZA and decitabine is recommended. In this regard, MDS have been well defined as an example of neoplastic HSC disease with particular chromosome abnormalities and a characteristic genomic landscape at the bulk analyses level (13, 14, 21–23), but the occurrence of mutational events at the single-cell level has only been recently delineated (25–27). However, these important studies have focused mostly on the dissection of the single-cell genetic events at diagnosis or addressing the progression to AML (25–27). Our work, beyond expanding the previous research avenues, has used a different angle, wondering whether the single-cell mutational and protein expression profile of the BM of these patients might also confer biomarker value to HMA treatment. The MDS single-cell portrait suggests that HMA responder patients undergo a depletion of mutant clones of progenitor and myeloid cells, such as HSPCs, immature granulocytes, and immature monocytes. The reduction of mutant HSPCs upon HMA treatment was further found associated with HI.

Our study constitutes one of the first attempts to delineate at the single-cell level both genetic and protein marks upon HMA treatment in MDS, but we acknowledge some of the limitations of the research. To obtain more definitive data with the capacity to predict HMA response in MDS, a much larger number of cases and additional timepoints would be necessary. In addition, due to the scarce material, we were not able to validate the surface protein composition using other methods such as flow cytometry. Fortunately, other authors have cross-validated the single-cell protein data with the same technology herein used with flow cytometry results (43, 46, 47). Finally, although the scDNA-seq approach is able to measure mutational zygosity, identify co-occurrence of mutational events and detect rare populations that could escape NGS analyses; not all mutations detected by NGS in our MDS cases were considered in our analysis. In this regard, despite the high correlation between the mutational events detected by scDNA-seq and NGS (Spearman correlation test *R* = 0.88; *P* = 1.6 e^−09^; Supplementary Fig. S1B), some mutations were not present in scDNA-seq for technical issues related to amplicon coverage and/or GC content of the amplified regions.

The only potential curative option for high-risk MDS would be, for those eligible, the allogeneic hematopoietic stem cell transplantation (allo-HSCT; refs. 44, 45). In this regard, the use of HMA has increased the survival of these patients, but beyond those individuals that already harbor from the beginning resistance to these drugs, those that are initially sensitive might become resistant to the epigenetic compound during the natural history of the disease (44, 45). However, if these original or new HMA refractory patients are still not immediately eligible for allo-HSCT, the longitudinal analyses of their immunophenotype and mutational context at the single-cell level, such as herein identified, could be useful to decide a second-line treatment. In this regard, the herein obtained single-cell mutant trajectories of MDS upon HMA treatment can also unveil eventually targetable clones in nonresponder patients. For example, in the identified *NRAS* mutant clones, the use of the drug rigosertib (48), which blocks the interaction of RNA and its effectors, could be tried. Interestingly, a small clinical trial using this compound in MDS after HMA failure did not observe survival improvement, but tailored treatment according to the load of the *RAS* mutant clone was not assessed. Furthermore, the identification by our scDNA-seq approach of the common co-occurrence of mutations from different pathways in the same cell suggest the potential use of combined target therapies. One example could be molecularly-oriented trials combining RAS inhibitors with molecules that preferentially kill spliceosome-mutant hematopoietic cells, such as the SF3B modulator H3B-8800 (49). A similar scenario can be drawn for the mutant *NF1* clones observed in HMA nonresponder cases, where MEK inhibitors, already studied in AML (50), could be also assessed alone or in combination with splicing inhibitors in clones with co-occurrent mutations for both pathways.

Beyond providing the distinct clonal cellular repertoire of HMA responders versus nonresponders, the determination of surface proteins at single-cell resolution, as herein performed, provides a meticulous quantification of clones harboring these potential targets for antibody therapies in MDS. Importantly for future developments in the field of novel agents for MDS, the herein used single-cell platform can incorporate those surface proteins where current clinical trials are underway and, in this manner, programmed death-1 (PD-1) receptor and ligand, CTLA4, TIM-3, CSF1R, or CD47 are amenable candidates (20). Related to this, the CD47-blocking mAb magrolimab, has recently shown promising efficacy in higher-risk MDS when combined with AZA (51).

Finally, the uniqueness of the herein used single-cell analyses that combines not only the detection of coexisting mutational events in the same cell, but also provides cellular type identity to that clone, represents an additional advantage to select therapies in HMA refractory patients with MDS. Beyond the simultaneous targeting of co-occurrent mutations in the progenitor myeloid clones in nonresponders, the obtained single-cell multiomics landscape provides new clues about how to interfere with other MDS-related pathways such as inflammation. It is thought that MDS arise and progress in a BM environment of enhanced proinflammatory signaling (52). Related to this proinflammatory milieu that drives the expansion of HSPCs, we have observed that the reduction of nonclassical monocytes is associated with the clinical response to the HMA regimen. Nonclassical monocytes are anti-inflammatory cells that are elevated and functionally impaired in high-risk MDS (53, 54). Thus, their disbalance might be associated with an overactivation of the proinflammatory signals that will further foster tumor immune escape. Consequently, it is tempting to propose that a subset of patients with HMA-resistant MDS could be more sensitive to the use of inhibitors of inflammation such as those targeting IRAK4 (Emavusertib) or TLR2 (Tomaralimab), both of them undergoing clinical trials in MDS (20). These HMA nonresponder patients could also be excellent candidates to assess the efficacy of immune checkpoint inhibitors such as pembrolizumab, nivolumab, and atezolizumab that are also under clinical trial assessment in MDS (20). In addition, and most importantly, in all these cases with refractoriness to HMA treatment, we could add the specific inhibitor(s) for the mutation(s) carried in that clone of the proinflammatory cell, such as the above described RAS and splicing inhibitors to stir up a stronger clinical response.

In summary, our results provide an insightful view at single-cell resolution of the dynamics at the genetic and protein level that takes place in the BM of patients with MDS upon HMA treatment. Our multiomics approach, beyond providing clues about the natural history of the disease, and its modification by using the epigenetic compound, sheds light on potential biomarkers associated with therapy efficacy. Related to the almost 50% of MDS cases that do not respond to HMAs (16, 55), our data suggest that these patients are characterized by complex mutational trajectories and persistence of mutant clones, particularly in HSPCs and specific myeloid cell populations. The particular genetic and immunophenotypic configuration could be vulnerable to the right combination of small drugs and antibodies tailored for each patient at the end of the HMA treatment. A personalized therapy could eventually lead to the clinical improvement of these patients that currently have a dismal outcome and no approved second-line therapies.

## Supplementary Material

Figure S1 Mutational burden at diagnosis across patients and correlation between single-cell and bulk VAFs

Figure S2 Distribution of mutations in CHIP-associated genes at diagnosis according to response status

Figure S3 Clonal landscape of MDS patients

Figure S4 Integration of all MDS patient samples and distribution of cell surface markers across cell populations

Figure S5 Impact of AZA treatment on cell populations according to response status

Figure S6 Differences in cell type abundance upon AZA treatment according to hematological improvement (HI) and marrow complete response (mCR)

Figure S7 Clonal landscape of the progenitor, immature erythroid, myeloid and lymphoid compartments at diagnosis for each patient

Figure S8 Distribution of WT and mutant cells per gene

Figure S9 Mutational status of the lymphoid compartment

Figure S10 Effect of AZA treatment on wild-type cell populations in responder patients

Figure S11 Impact of AZA treatment on mutant cell populations in patients with hematological improvement (HI)

Table S1 Clinicopathologic information of the studied MDS patients

Table S2 Amplicon coverage for the custom single cell DNA sequencing panel

Table S3 Antibodies used for single cell surface protein sequencing

Table S4 Allele dropout (ADO) rate per sample (diagnosis and after AZA treatment)

Table S5 Fisher exact test for each mutated gene, comparing responders vs. non-responders at diagnosis

Table S6 Karyotype and mutations (bulk NGS and single-cell DNAseq) at diagnosis for the studied MDS cases

Table S7 Fisher exact test for each mutated pathway, comparing responders vs. non-responders at diagnosis

Table S8 Fisher exact test for each combination of mutated pathways, comparing responders vs. non-responders at diagnosis

Table S9 Fisher exact test of total cells from patients with each mutated gene, comparing mutant and wild-type (WT) progenitor-immature erythroid-myeloid (Prog_Ery_Mye) and lymphoid compartments
