# Supplementary material for: Single-cell Multiomics Analysis of Myelodysplastic Syndromes and Clinical Response to Hypomethylating Therapy
Source: Cancer Res Commun. 2024 Feb 12;4(2):365–77. doi: 10.1158/2767-9764.CRC-23-0389 (PMC10860538; doi:10.1158/2767-9764.CRC-23-0389)
Supplement: Figure S1 — Mutational burden at diagnosis across patients and correlation between single-cell and bulk VAFs [file crc-23-0389-s01.pdf]

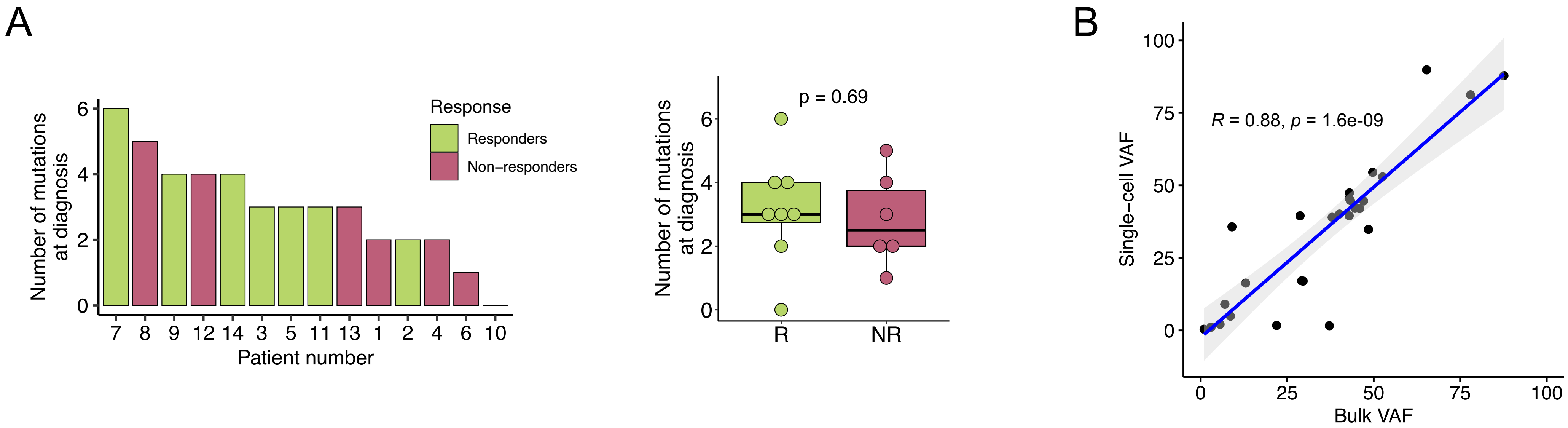

Supplementary Figure 1. Mutational burden at diagnosis across patients and correlation between single-cell and bulk VAFs. A. Number of mutations per patient at diagnosis, colored by response status (left); Number of mutations in responders (R) and non-responders (NR) at diagnosis (right). B. Spearman correlation between Tapestri variant allele frequency (VAF) by read count and bulk sequencing VAF of common variants.
