# Supplementary material for: Single-cell Multiomics Analysis of Myelodysplastic Syndromes and Clinical Response to Hypomethylating Therapy
Source: Cancer Res Commun. 2024 Feb 12;4(2):365–77. doi: 10.1158/2767-9764.CRC-23-0389 (PMC10860538; doi:10.1158/2767-9764.CRC-23-0389)
Supplement: Figure S2 — Distribution of mutations in CHIP-associated genes at diagnosis according to response status [file crc-23-0389-s02.pdf]

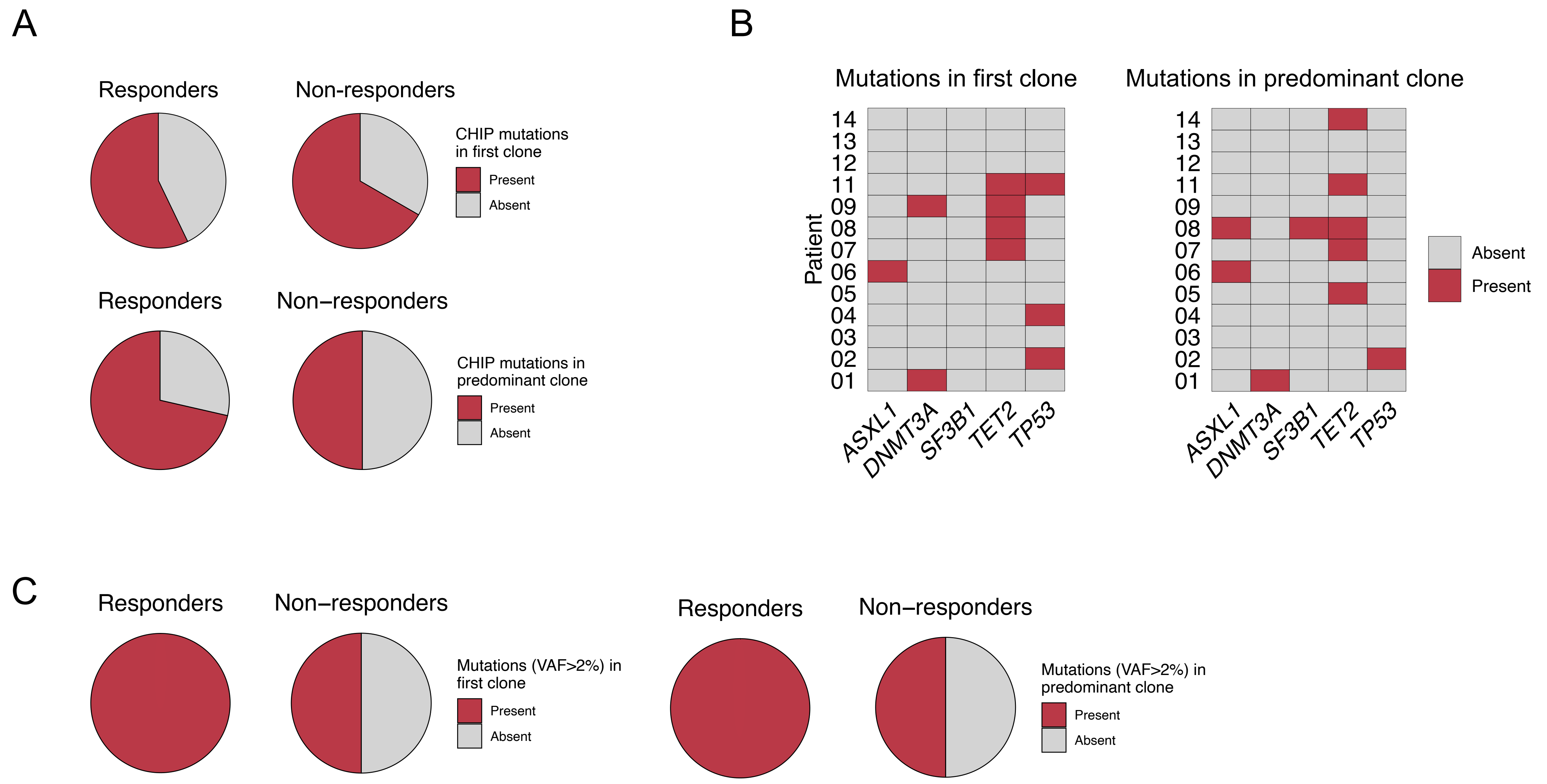

Supplementary Figure 2. Distribution of mutations in CHIP-associated genes at diagnosis according to response status. A. Proportion of responder and non-responder patients with mutations in CHIP-associated genes (*DNMT3A, TET2, ASXL1, SF3B1* and *TP53*) in first (upper) and predominant (bottom) clone at diagnosis (Fisher exact test, non-significant). B. Mutated CHIP-associated genes (*DNMT3A, TET2, ASXL1, SF3B1* and *TP53*) in each patient in first (left) and predominant (right) clone at diagnosis (patient #10 is not shown as no mutations were found). C. Proportion of responder and non-responder patients with mutations with VAF>2% in first (left) and predominant (right) clone at diagnosis (Fisher exact test, non-significant).
