# Supplementary material for: Single-cell Multiomics Analysis of Myelodysplastic Syndromes and Clinical Response to Hypomethylating Therapy
Source: Cancer Res Commun. 2024 Feb 12;4(2):365–77. doi: 10.1158/2767-9764.CRC-23-0389 (PMC10860538; doi:10.1158/2767-9764.CRC-23-0389)
Supplement: Figure S3 — Clonal landscape of MDS patients [file crc-23-0389-s03.pdf]

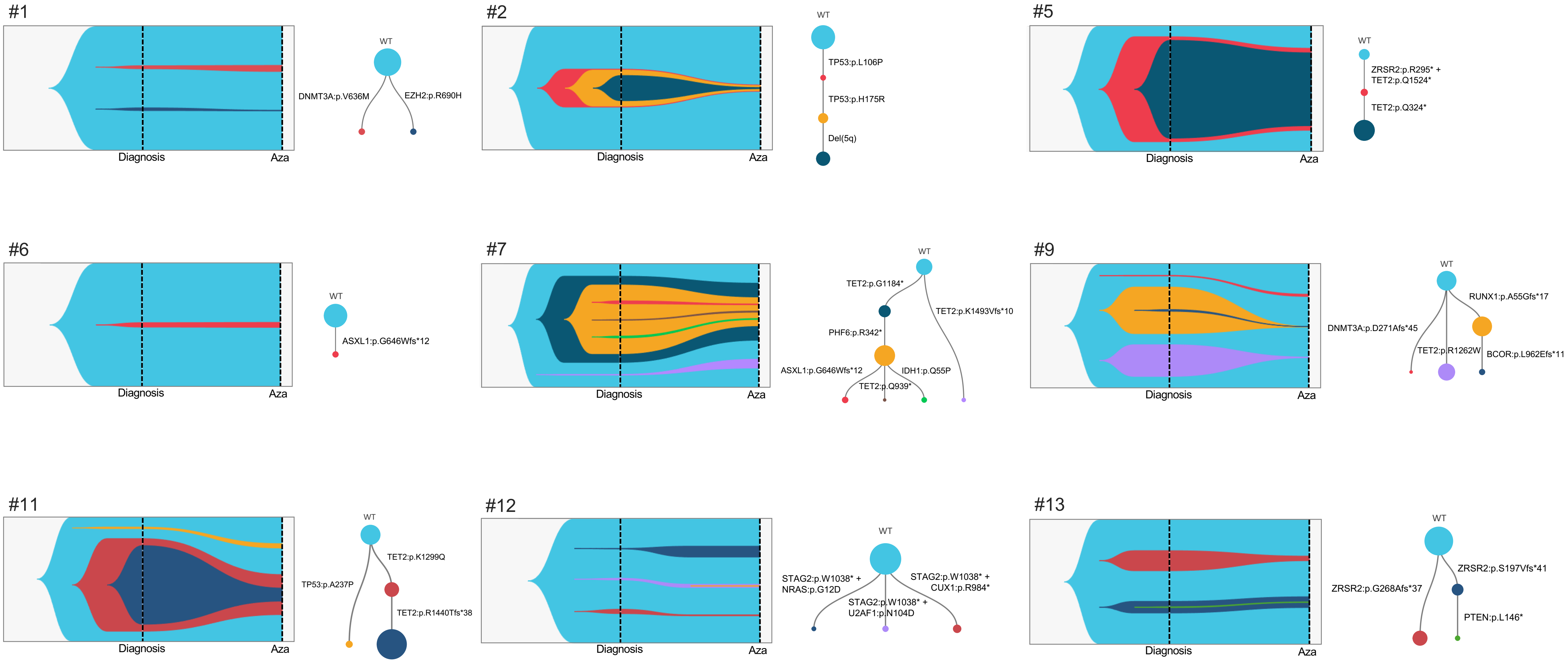

Supplementary Figure 3. Clonal landscape of MDS patients. Fishplots of patients #1, #2, #5, #6, #7, #9, #11, #12 and #13 illustrating the clonal distribution at diagnosis and after AZA treatment, and their clonal phylogenies at diagnosis. Patient #10 is not shown as no mutations were identified.
