# Supplementary material for: Single-cell Multiomics Analysis of Myelodysplastic Syndromes and Clinical Response to Hypomethylating Therapy
Source: Cancer Res Commun. 2024 Feb 12;4(2):365–77. doi: 10.1158/2767-9764.CRC-23-0389 (PMC10860538; doi:10.1158/2767-9764.CRC-23-0389)
Supplement: Figure S4 — Integration of all MDS patient samples and distribution of cell surface markers across cell populations [file crc-23-0389-s04.pdf]

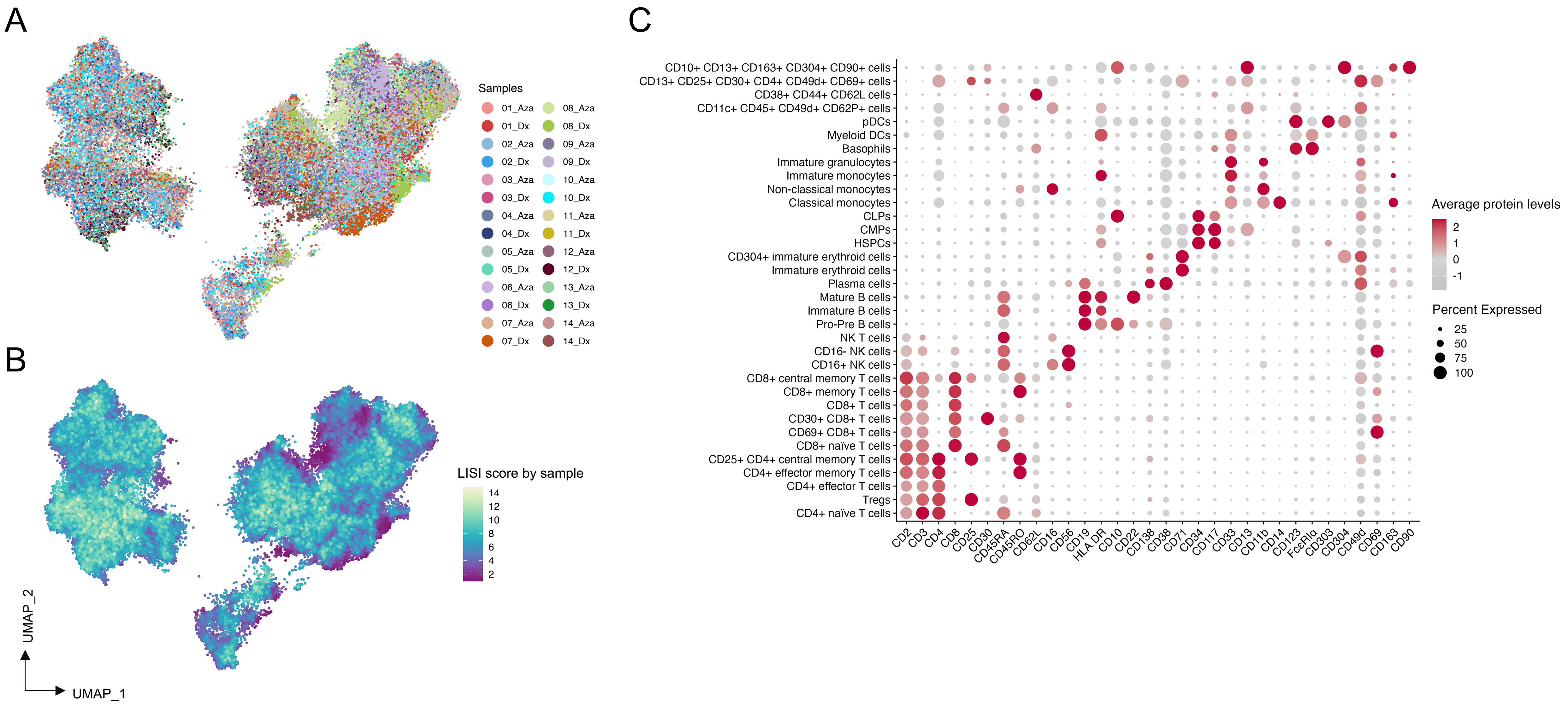

Supplementary Figure 4. Integration of all MDS patient samples and distribution of cell surface markers across cell populations. A. UMAP visualization colored by sample. Dx, diagnosis; Aza, after AZA treatment. B. UMAP visualization colored by LISI (Local Inverse Simpson's Index) score computed by sample. C. Dot plot showing the abundance of the cell surface makers used for the annotation of the cell populations.
