# Supplementary material for: Single-cell Multiomics Analysis of Myelodysplastic Syndromes and Clinical Response to Hypomethylating Therapy
Source: Cancer Res Commun. 2024 Feb 12;4(2):365–77. doi: 10.1158/2767-9764.CRC-23-0389 (PMC10860538; doi:10.1158/2767-9764.CRC-23-0389)
Supplement: Figure S5 — Impact of AZA treatment on cell populations according to response status [file crc-23-0389-s05.pdf]

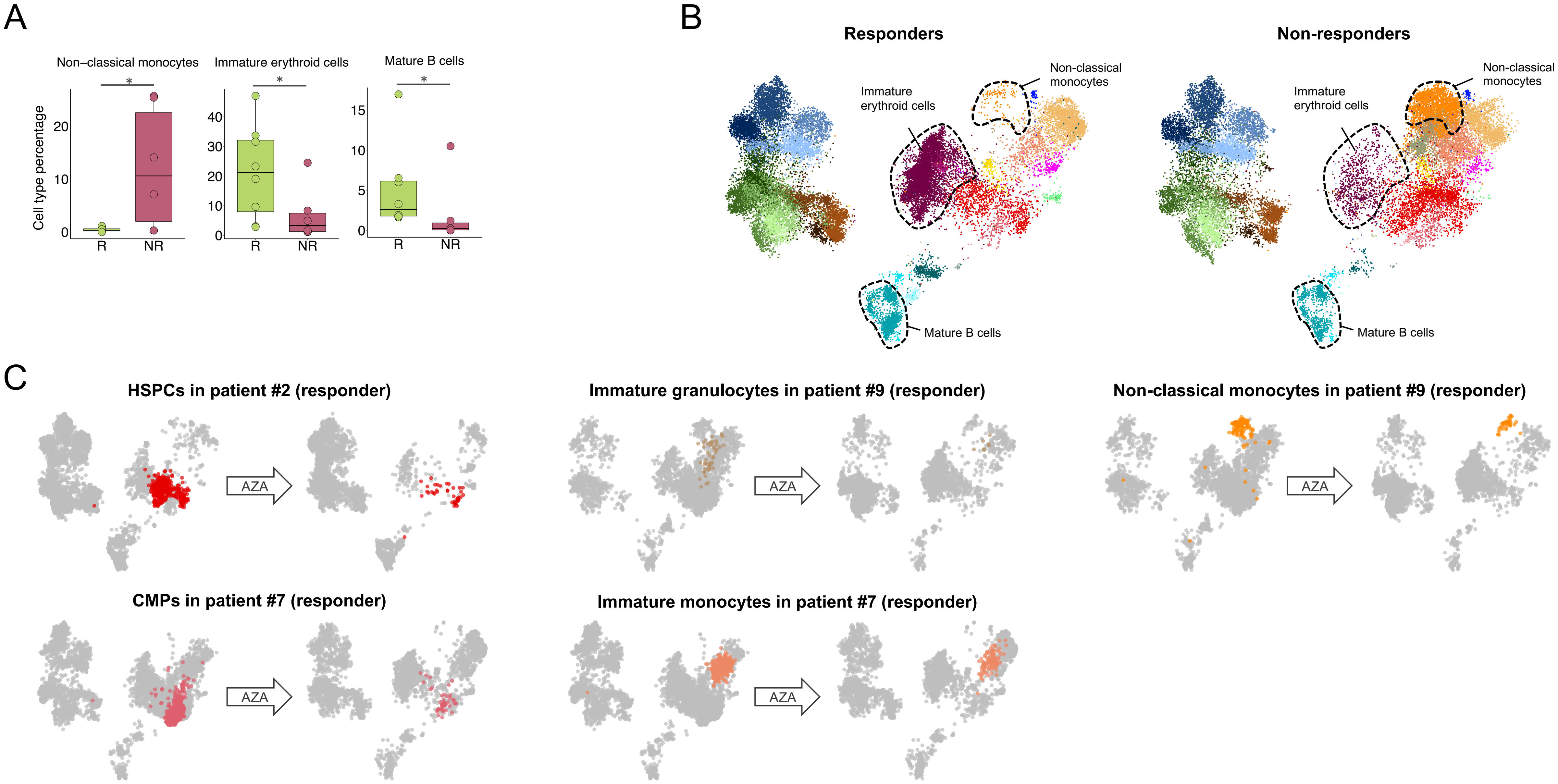

Supplementary Figure 5. Impact of AZA treatment on cell populations according to response status. A. Cell type proportions in responders and non-responders after AZA treatment. *, scCODA FDR<0.1. B. UMAP visualization of responders (8 patients) and non-responders (6 patients) after AZA treatment colored by cell type (color scheme corresponding to Figure 4B), highlighting cell populations indicated in panel A. C. UMAP visualizations highlighting HSPCs in patient #2, CMPs in patient #7, immature granulocytes in patient #9, immature monocytes in patient #7 and non-classical monocytes in patient #9, at diagnosis and after AZA treatment. R, responders; NR, non-responders.
