# Supplementary material for: Single-cell Multiomics Analysis of Myelodysplastic Syndromes and Clinical Response to Hypomethylating Therapy
Source: Cancer Res Commun. 2024 Feb 12;4(2):365–77. doi: 10.1158/2767-9764.CRC-23-0389 (PMC10860538; doi:10.1158/2767-9764.CRC-23-0389)
Supplement: Figure S6 — Differences in cell type abundance upon AZA treatment according to hematological improvement (HI) and marrow complete response (mCR) [file crc-23-0389-s06.pdf]

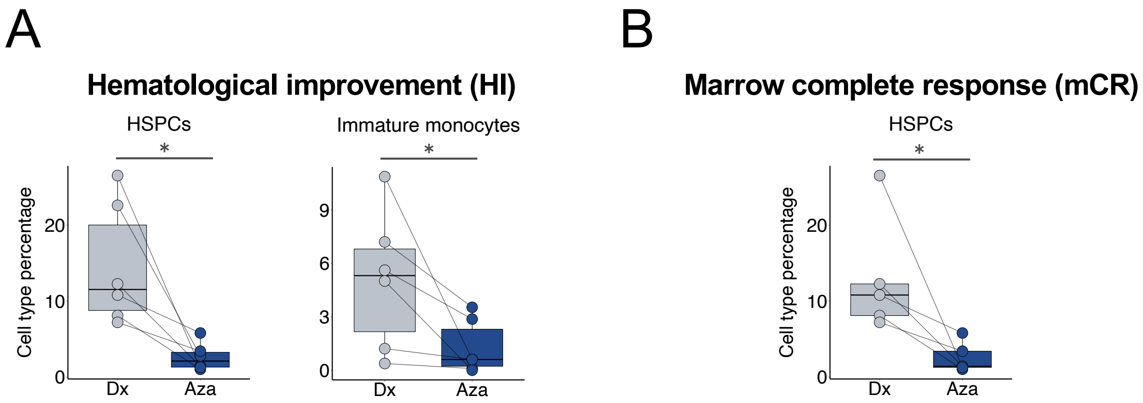

Supplementary Figure 6. Differences in cell type abundance upon AZA treatment according to hematological improvement (HI) and marrow complete response (mCR). A. Cell type proportions at diagnosis and after AZA treatment in patients with HI. B. Cell type proportions at diagnosis and after AZA treatment in patients with mCR. *, scCODA FDR<0.1; Dx, Diagnosis; Aza, After AZA treatment.
