# Supplementary material for: Single-cell Multiomics Analysis of Myelodysplastic Syndromes and Clinical Response to Hypomethylating Therapy
Source: Cancer Res Commun. 2024 Feb 12;4(2):365–77. doi: 10.1158/2767-9764.CRC-23-0389 (PMC10860538; doi:10.1158/2767-9764.CRC-23-0389)
Supplement: Figure S7 — Clonal landscape of the progenitor, immature erythroid, myeloid and lymphoid compartments at diagnosis for each patient [file crc-23-0389-s07.pdf]

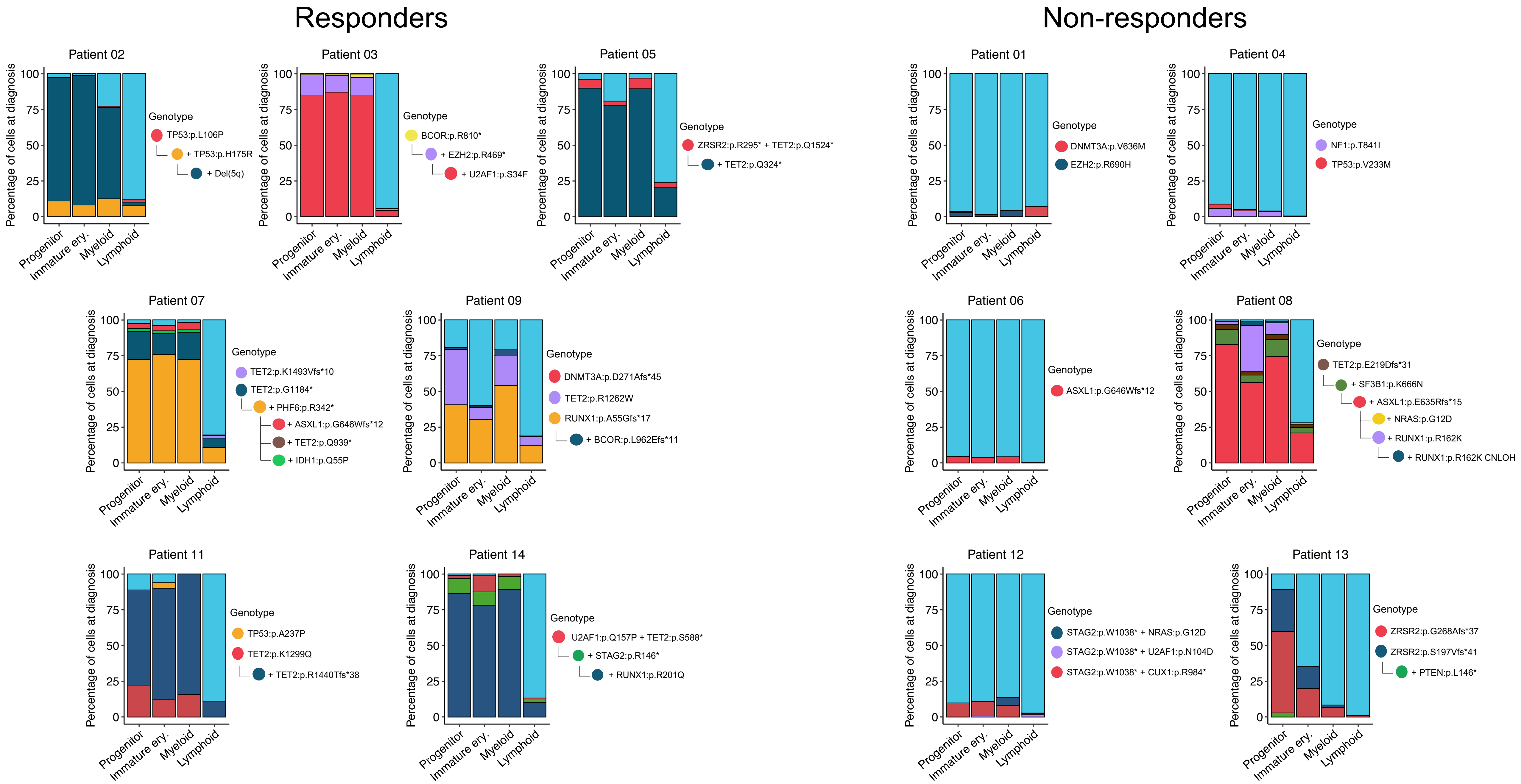

Supplementary Figure 7. Clonal landscape of the progenitor, immature erythroid, myeloid and lymphoid compartments at diagnosis for each patient. Color scheme of the clones relates to phylogenies and fishplots (Figure 3 and Supplementary Figure 3). Light blue represents WT. Patient #10 is not shown as no mutations were identified.
