# Supplementary material for: Single-cell Multiomics Analysis of Myelodysplastic Syndromes and Clinical Response to Hypomethylating Therapy
Source: Cancer Res Commun. 2024 Feb 12;4(2):365–77. doi: 10.1158/2767-9764.CRC-23-0389 (PMC10860538; doi:10.1158/2767-9764.CRC-23-0389)
Supplement: Figure S8 — Distribution of WT and mutant cells per gene [file crc-23-0389-s08.pdf]

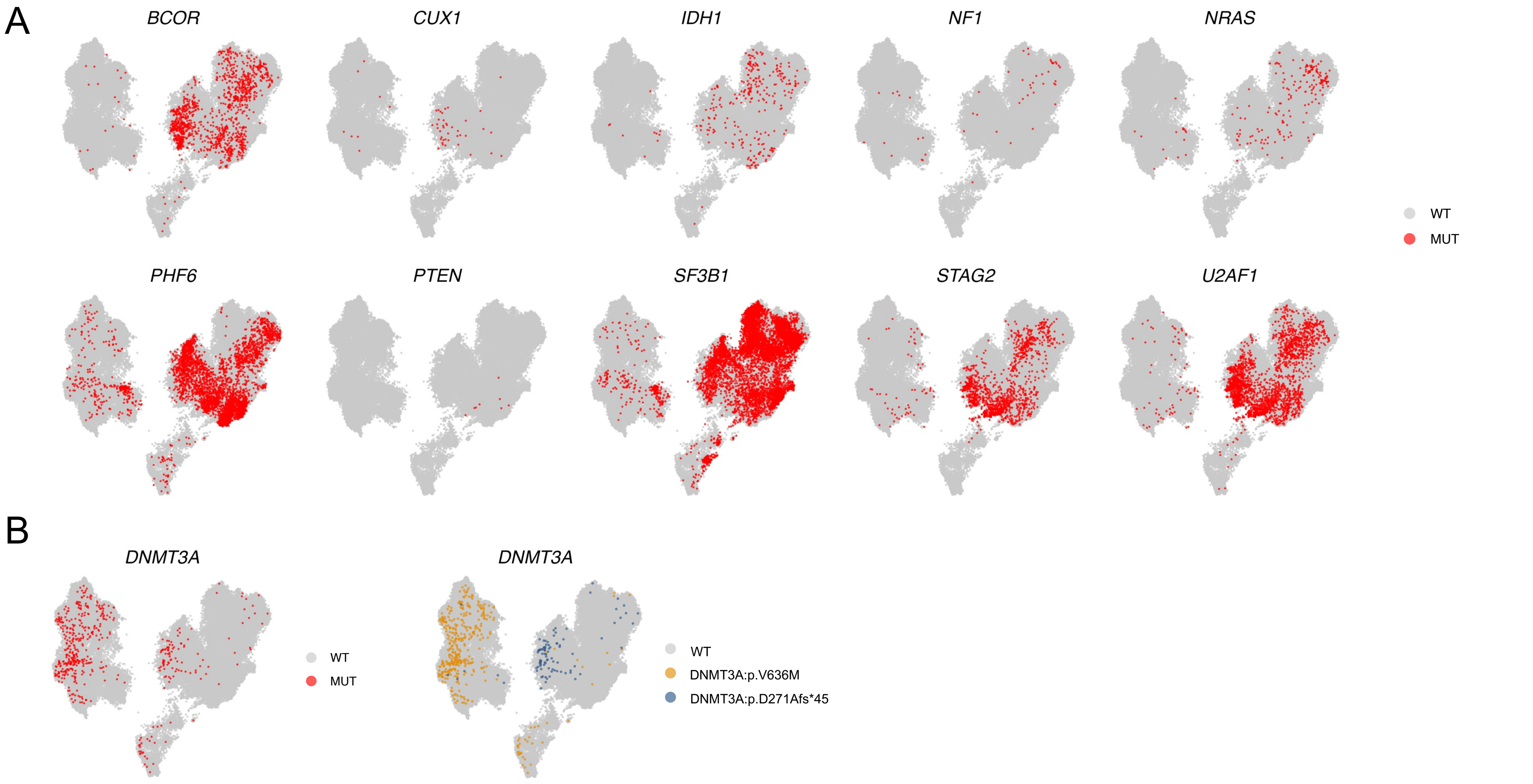

Supplementary Figure 8. Distribution of WT and mutant cells per gene. A. UMAP visualization of all bone marrow cells colored by mutational status of each gene. B. UMAP visualization of DNMT3A colored by mutation status (left); UMAP visualization showing DNMT3A mutations colored by coding impact (right). WT, wild-type; MUT, mutant.
