# Supplementary material for: Single-cell Multiomics Analysis of Myelodysplastic Syndromes and Clinical Response to Hypomethylating Therapy
Source: Cancer Res Commun. 2024 Feb 12;4(2):365–77. doi: 10.1158/2767-9764.CRC-23-0389 (PMC10860538; doi:10.1158/2767-9764.CRC-23-0389)
Supplement: Figure S9 — Mutational status of the lymphoid compartment [file crc-23-0389-s09.pdf]

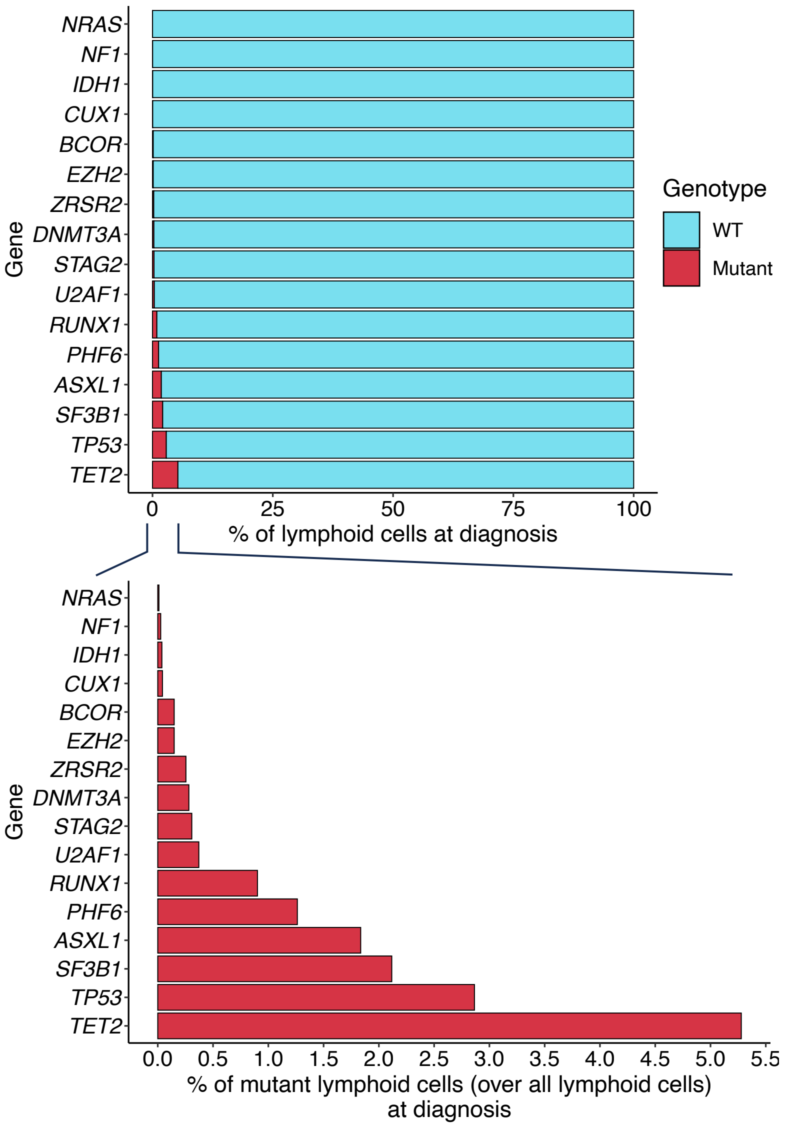

Supplementary Figure 9. Mutational status of the lymphoid compartment. Percentage of mutant lymphoid cells (over all lymphoid cells) for each gene at diagnosis.
