# Supplementary material for: Single-cell Multiomics Analysis of Myelodysplastic Syndromes and Clinical Response to Hypomethylating Therapy
Source: Cancer Res Commun. 2024 Feb 12;4(2):365–77. doi: 10.1158/2767-9764.CRC-23-0389 (PMC10860538; doi:10.1158/2767-9764.CRC-23-0389)
Supplement: Figure S10 — Effect of AZA treatment on wild-type cell populations in responder patients [file crc-23-0389-s10.pdf]

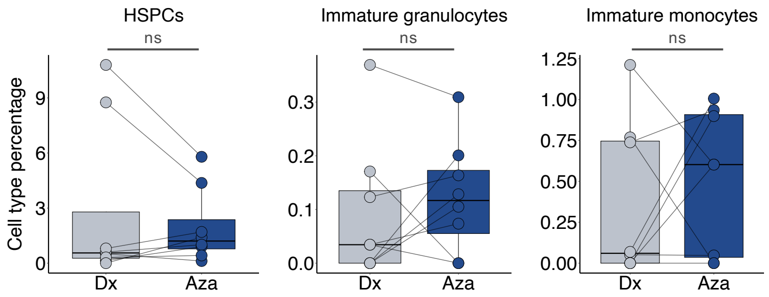

Supplementary Figure 10. Effect of AZA treatment on wild-type cell populations in responder patients. Dx, diagnosis; Aza, after AZA treatment; ns, non-significant; scCODA FDR>0.1
