# Supplementary material for: Single-cell Multiomics Analysis of Myelodysplastic Syndromes and Clinical Response to Hypomethylating Therapy
Source: Cancer Res Commun. 2024 Feb 12;4(2):365–77. doi: 10.1158/2767-9764.CRC-23-0389 (PMC10860538; doi:10.1158/2767-9764.CRC-23-0389)
Supplement: Figure S11 — Impact of AZA treatment on mutant cell populations in patients with hematological improvement (HI) [file crc-23-0389-s11.pdf]

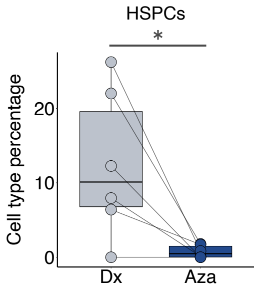

Supplementary Figure 11. Impact of AZA treatment on mutant cell populations in patients with hematological improvement (HI). Mutant cell type proportion at diagnosis (Dx) and after AZA treatment (Aza) in patients with HI. *, scCODA FDR<0.1.
